# Resource competition explains rare cannibalism in the wild in livebearing fishes

**DOI:** 10.1002/ece3.8872

**Published:** 2022-05-16

**Authors:** Rüdiger Riesch, Márcio S. Araújo, Stuart Bumgarner, Caitlynn Filla, Laura Pennafort, Taylor R. Goins, Darlene Lucion, Amber M. Makowicz, Ryan A. Martin, Sara Pirroni, R. Brian Langerhans

**Affiliations:** ^1^ 3162 Department of Biological Sciences Centre for Ecology, Evolution and Behaviour Royal Holloway University of London Egham UK; ^2^ Instituto de Biociências Universidade Estadual Paulista (UNESP) Rio Claro Brazil; ^3^ 6798 Department of Biological Sciences North Carolina State University Raleigh North Carolina USA; ^4^ Department of Anthropology University of Florida Gainesville Florida USA; ^5^ 7823 Department of Biological Sciences Florida State University Tallahassee Florida USA; ^6^ 2546 Department of Biology Case Western Reserve University Cleveland Ohio USA

**Keywords:** *Gambusia*, intraspecific competition, optimal foraging theory, Poecilia reticulata, population density, size difference

## Abstract

Cannibalism, the act of preying on and consuming a conspecific, is taxonomically widespread, and putatively important in the wild, particularly in teleost fishes. Nonetheless, most studies of cannibalism in fishes have been performed in the laboratory. Here, we test four predictions for the evolution of cannibalism by conducting one of the largest assessments of cannibalism in the wild to date coupled with a mesocosm experiment. Focusing on mosquitofishes and guppies, we examined 17 species (11,946 individuals) across 189 populations in the wild, spanning both native and invasive ranges and including disparate types of habitats. We found cannibalism to be quite rare in the wild: most populations and species showed no evidence of cannibalism, and the prevalence of cannibalism was typically less than 5% within populations when it occurred. Most victims were juveniles (94%; only half of these appeared to have been newborn offspring), with the remaining 6% of victims being adult males. Females exhibited more cannibalism than males, but this was only partially explained by their larger body size, suggesting greater energetic requirements of reproduction likely play a role as well. We found no evidence that dispersal‐limited environments had a lower prevalence of cannibalism, but prevalence was greater in populations with higher conspecific densities, suggesting that more intense resource competition drives cannibalistic behavior. Supporting this conclusion, our mesocosm experiment revealed that cannibalism prevalence increased with higher conspecific density and lower resource levels but was not associated with juvenile density or strongly influenced by predation risk. We suggest that cannibalism in livebearing fishes is rare in the wild because preying on conspecifics is energetically costly and only becomes worth the effort when competition for other food is intense. Due to the artificially reduced cost of capturing conspecifics within confined spaces, cannibalism in captive settings can be much more frequent.

## Graphical abstract

We assessed the occurrence of cannibalism in mosquitofishes (genus *Gambusia*) and guppies (*Poecilia reticulata*) by examining 17 species (11,946 individuals) across 189 populations in the wild, and by conducting a mesocosm experiment for one species (*Gambusia affinis*). Cannibalism was rare, its occurrence was associated with resource competition, but it was not associated with juvenile density or strongly influenced by predation risk, and cannibals were predominantly female. Cannibalism in captive settings might be higher due to an artificially reduced cost of capturing conspecifics within confined spaces.

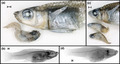

## 1 INTRODUCTION

Cannibalism describes the act of one individual preying on and (partially or completely) consuming another individual of the same species. Why individuals of certain species, including humans, resort to this extreme behavior has caught the attention and imagination of laypeople and scientists for centuries (e.g., Alighieri, 2013; Bailey, 2017; Bygott, 1972; Defoe, 1998; Hancock, 1852; Harner, 1977; Mead et al., 2003; White, 2001). Initially, scientists considered cases of cannibalism as behavioral abnormalities (e.g., Denenberg et al., 1959; Eible‐Eibelsfelt, 1961; Lapage, 1922), but this view shifted toward an understanding that cannibalism occurs in natural communities, is taxonomically widespread, is influenced by natural selection just like other behaviors, and can have important ecological and evolutionary consequences (e.g., Bailey, 2017; Elgar & Crespi, 1992; Fedurek et al., 2020; Fox, 1975; Ibáñez & Keyl, 2010; Manica, 2002; Pereira et al., 2017; Polis, 1981; Richardson et al., 2010; Rudolf, 2007). For instance, cannibalism has often been implicated as an important mechanism of population regulation in natural populations (e.g., Houghton et al., 2017; Ricker, 1954; Van Buskirk, 1989) and may facilitate colonization of new environments and population persistence through stressful periods (e.g., Agarwala & Dixon, 1992; Via, 1999; Watanabe & Yamaguchi, 1993). But how selection shapes the prevalence of cannibalism in different taxa remains a major outstanding question.

Under adaptive evolution, cannibalism should generally evolve according to the prevailing costs and benefits of the behavior (e.g., Boots et al., 2021; Mitchell & Walls, 2008; Pfennig, 1997; Rudolf et al., 2010). Variation in these costs and benefits among species, populations, and individuals may largely explain the extensive variation in cannibalism prevalence that occurs in nature (e.g., Manica, 2002; Nilsson et al., 2011; Parsons et al., 2013). The special case of sexual cannibalism (i.e., one sexual partner consuming the other) can involve unique costs and benefits associated with sexual conflict (e.g., Andrade, 1996; Boisseau et al., 2017; Elgar & Schneider, 2004; Schneider, 2014; Schwartz et al., 2016; Wilder & Rypstra, 2008), but here we focus on other forms of cannibalism. The benefits of cannibalism can be strong, as conspecifics can provide high‐quality food (e.g., Agarwala & Dixon, 1992; Meffe & Crump, 1987; Mehlis et al., 2009; Via, 1999) and cannibalism can remove potential competitors (e.g., Elgar & Crespi, 1992; Klug & Bonsall, 2019; Polis, 1981). However, there are several potential costs of cannibalism. First, cannibalizing relatives, such as one's own offspring (i.e., filial cannibalism), can reduce total fitness (Hamilton, 1964a, 1964b,1964a, 1964b; Pfennig, 1997). Kin recognition, and biased cannibalization of non‐relatives, can alleviate this cost (Pfennig & Collins, 1993; Pfennig et al., 1993). Second, cannibalization can require high energetic expenditure or particular/specialized traits to locate, capture, or consume conspecific prey relative to alternative prey (Baras et al., 2010; Pfennig, 1992). For instance, conspecific prey can present special challenges compared to more typical prey, such as more cryptic behaviors or morphologies, larger body sizes, or enhanced or divergent escape abilities (e.g., locomotor performance, morphological defenses; Collins & Cheek, 1983; Pfennig, 1992; Williamson & Vanderploeg, 1988; Yasuda et al., 2001). Third, cannibalism could facilitate parasite and pathogen transmission (Pfennig et al., 1998; Sadeh et al., 2016; but see Rudolf & Antonovics, 2007; Van Allen et al., 2017). Fourth, pursuing conspecific prey can increase vulnerability to predators relative to alternative foraging behaviors, for example, if cannibalistic behaviors reduce vigilance or draw greater attention from predators owing to altered behaviors or other phenotypes (Fernández‐Juricic & Tran, 2007; Milinski & Heller, 1978).

Cannibalism may often be plastic if these costs and benefits vary predictably over relevant temporal and spatial scales, such as facultative cannibalism when alternative prey are relatively scarce—increased cannibalism under higher densities or hunger has been demonstrated for diverse taxa and from both experimental and natural populations (Barkae et al., 2014; Fox, 1975; Naseer & Abdurahiman, 1993; Petersen et al., 2010; Polis, 1981). Costs and benefits of cannibalism can also vary among individuals (e.g., sex, morph) or within an individual's life based on developmental stage, reproductive status, or body size (Colchen et al., 2019; Elwood, 1994; Hubbs, 1991; Lewis et al., 2010; Manica, 2002; Parsons et al., 2013; Pfennig, 1997; Schausberger, 2003). Altogether, cannibalism prevalence should vary widely among taxa—and even among populations and individuals—based on these costs and benefits, with the highest frequencies observed when the relative benefits are highest and costs are lowest, such as when avoidance of relatives is easily accomplished, alternative prey are scarce or low‐quality, predation risk is low, and conspecific prey are highly nutritious and readily encountered, captured, and consumed.

Inter‐ and intracohort cannibalism (i.e., cannibalism between and within the same cohort/generation) are widespread in teleost fishes and have been described for both marine and freshwater taxa (Manica, 2002; Mitchell & Walls, 2008; Pereira et al., 2017; Smith & Reay, 1991). One group, the livebearing fishes of the family Poeciliidae, has long been known for the (what appears to be common) occurrence of cannibalism, which has been reported from many different genera (e.g., *Belonesox*, *Gambusia*, *Heterandria*, *Poecilia*, *Poeciliopsis*, and *Xiphophorus*; Meffe & Snelson, 1989; Manica, 2002; Pereira et al., 2017) and settings (wild populations: e.g., Nesbit & Meffe, 1993; Specziár, 2004; laboratory/experimental settings: e.g., Dionne, 1985; Hubbs & Schlupp, 2008; Meffe, 1984; Nilsson et al., 2011). In fact, this behavior often poses obstacles for breeding poeciliid fishes in aquaculture facilities, laboratory research, and the aquarium hobby (e.g., Baldwin, 1980; Barki et al., 2014; Jones et al., 1998; Naumowicz et al., 2017), and usually takes the form of filial cannibalism (i.e., parents consuming their own offspring) and nonparental cannibalism (i.e., individuals of an older generation cannibalizing unrelated younger conspecifics; Manica, 2002).

Different genera and species of livebearing fishes differ in their propensity for cannibalism, with mosquitofishes (genus *Gambusia*) and guppies (*Poecilia reticulata*) often at the forefront of cannibalism research in this family (e.g., Breder & Coates, 1932; Loekle et al., 1982; Manica, 2002; Meffe & Snelson, 1989; Nilsson & Persson, 2013; Pereira et al., 2017; Rose, 1959). Cannibalism rates vary among species and populations in these groups, and females typically show higher cannibalism rates than males (e.g., Hubbs, 1991, 1992, 1996; Nesbit & Meffe, 1993; Nilsson et al., 2011). However, the majority of cannibalism studies in these taxa—and most fishes—have focused on laboratory stocks or at least experimental settings in which rates of cannibalism may be much higher than naturally occur in the wild. For instance, experimental work has often reported high rates of cannibalism (e.g., Dionne, 1985; Hubbs, 1996; Nilsson et al., 2011; Nilsson & Persson, 2013), while dietary studies in the wild (not directly focused on cannibalism) have typically reported relatively rare occurrences of cannibalism (e.g., Crivelli & Boy, 1987; Gluckman & Hartney, 2000; Greenfield et al., 1983; Hubbs, 1971, 1991; Nesbit & Meffe, 1993; Rakocinski & Greenfield, 1985; Specziár, 2004; Zandonà et al., 2011; Zandonà et al., 2015; but see Remon et al., 2016). Another weakness of previous studies is the low level of replication in most cases, as the focus was usually on just a single (e.g., Dionne, 1985; Specziár, 2004) or a handful of populations of a single species (Crivelli & Boy, 1987; Nesbit & Meffe, 1993; Nilsson et al., 2011; but see Hubbs, 1991, 1996). Therefore, we still do not fully understand the prevalence of cannibalism in natural populations, or the ecological factors that may influence cannibalism in the wild. Without that knowledge, we cannot determine whether cannibalism represents a common or strong selective force that shapes phenotypes in the wild, or whether more care needs to be taken in experimental and captive settings to prevent high rates of cannibalism which might be uncharacteristic of natural settings.

**FIGURE 1:**
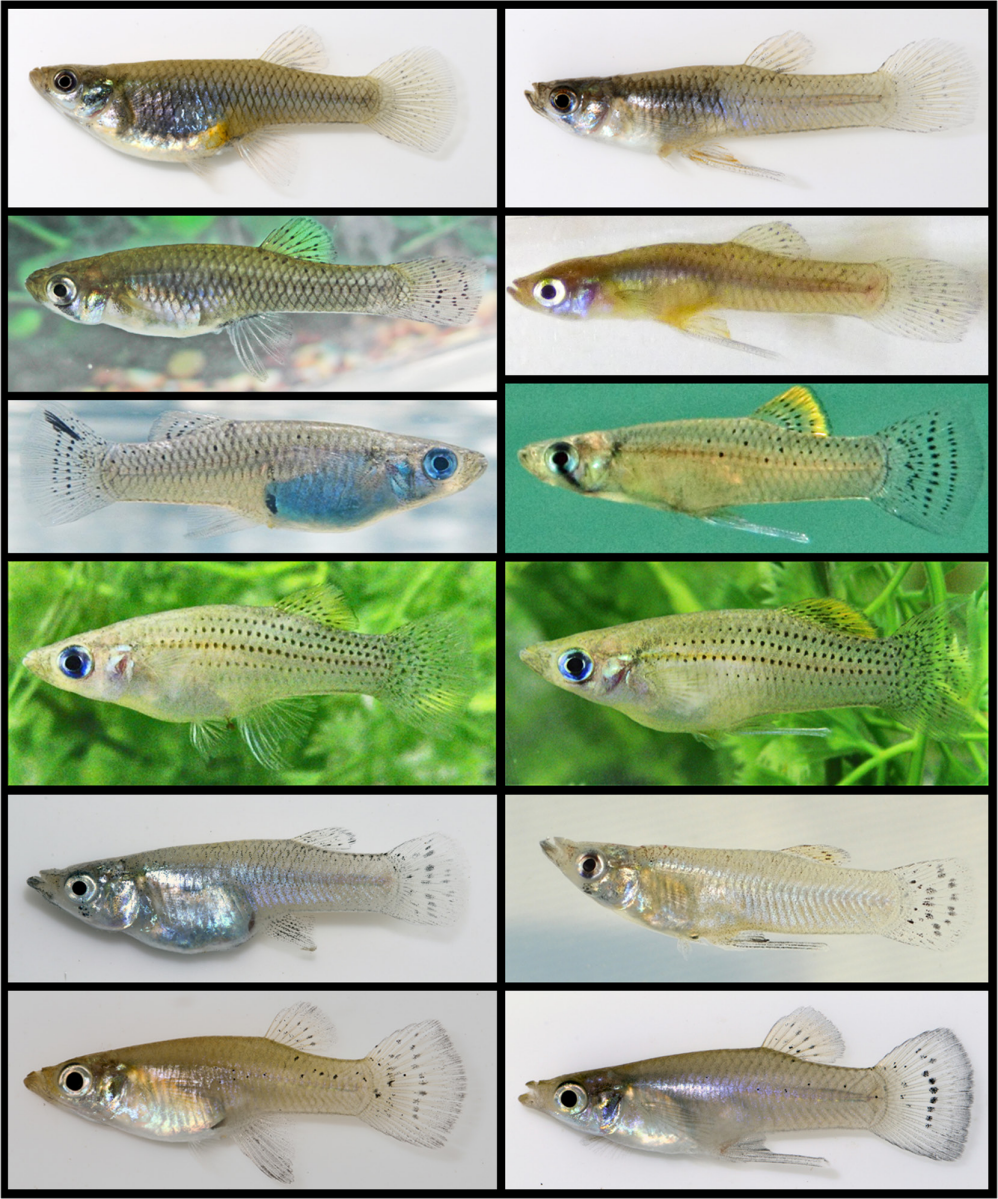
Example photographs of some of the studied species, with females on the left and males on the right; pictures not to scale. From the top: *Gambusia affinis*, *G*. *holbrooki*, *G*. *hubbsi*, *G*. *rhizophorae*, *G*. *eurystoma*, and *G*. *sexradiata*. *Gambusia affinis*, *G*. *eurystoma* and *G*. *sexradiata* photos taken by Michael Tobler, *G*. *holbrooki* photos taken by Rüdiger Riesch, and *G*. *hubbsi* and *G*. *rhizophorae* photos taken by R. Brian Langerhans

Here, we examine the largest dataset to date, to our knowledge, of cannibalism rates in the wild by focusing on 16 species of mosquitofishes (total *N* = 11,469; Figure 1) and on guppies (total *N* = 477) and conduct an outdoor mesocosm experiment with *Gambusia affinis* (Western mosquitofish) to assess both the prevalence of cannibalism in nature and the factors that explain variation in this behavior. We specifically test four predictions. Prediction 1: because conspecific individuals represent large and highly evasive prey relative to typical prey (even newborns are large and evasive compared to primary *Gambusia* prey of insects and crustaceans), we predicted that the relatively large energetic cost of cannibalism would result in (a) overall rarity of cannibalism in the wild, and (b) cannibalism to become more common under more intense resource competition (e.g., Barkae et al., 2014; Bartlett, 1987; Dionne, 1985; Hoffmann & Pfennig, 1999; Ibáñez & Keyl, 2010; Rose, 1959; Tayeh et al., 2014; Thibault, 1974; Vaissi & Sharifi, 2016). Prediction 2: because female mosquitofish have higher energy requirements than males—greater reproductive investment, indeterminate growth, larger body size—and generally exhibit a larger size difference between themselves and possible victims, we predicted that females would show higher rates of cannibalism than males (Claessen et al., 2004; Hubbs, 1991, 1992, 1996; Nesbit & Meffe, 1993; Nilsson et al., 2011). Prediction 3: because pursuing evasive conspecifics could increase vulnerability to predators relative to typical foraging behaviors (e.g., elevate visual detection, reduce vigilance), combined with potentially increased use of refuge by juveniles under high predation threat, we predicted that cannibalism rates would decrease under higher risk of predation (Benoît et al., 2000; Kishida et al., 2009; Kishida et al., 2011; Nilsson & Persson, 2013; but see Tigreros et al., 2017). Prediction 4: prior work has shown that cannibalism rates should decrease in populations with greater dispersal limitation (i.e., fewer emigrants) because individuals have a greater probability of cannibalizing kin in those situations (Boots et al., 2021; Lion & van Baalen, 2007; Rudolf et al., 2010). However, because poeciliid fish are known to employ self‐referent phenotype matching, can recognize kin, and can bias cannibalism toward non‐relatives (e.g., Greenway et al., 2016; Hain et al., 2017; Langerhans & Makowicz, 2013; Loekle et al., 1982), we predicted that cannibalism rates in the wild would not match those predictions.

## 2 METHODS

To test our four predictions, we used a three‐pronged approach involving a large field survey, comparative analyses of well‐studied natural populations, and a mesocosm experiment. We first conducted broad surveys of wild‐caught adult mosquitofishes and guppies to assess cannibalism rates in the wild across multiple populations of multiple species from native and introduced ranges across a variety of habitat types (e.g., ditches, rivers, ponds, lakes, toxic sulfur springs, asphalt lakes, blue holes, and marine environment; Table 1; Tables S1–S3). These surveys were designed to evaluate the overall prevalence of cannibalism in nature (prediction 1a) and test for differences in cannibalism prevalence between sexes (prediction 2). We focused exclusively on adults because we assumed cannibalism in juveniles would be especially rare considering their mouths and guts are smaller than most conspecific individuals, and the focal species rarely partially consume prey, but rather typically eat prey whole. We thus centered on cannibalism in the sense of killing and consuming whole individuals, not other cases such as fin nipping, scale eating, or scavenging parts of deceased conspecifics—these types of cannibalism are apparently extremely rare in the wild in these taxa because we never encountered such prey parts in any stomachs examined in this study. All fish examined in the surveys were collected using dip nets and seines, and immediately euthanized and preserved (95% ethanol or 10% formalin) to prevent further feeding or digestion. All collections occurred when small, young conspecifics (i.e., the most likely potential victims) were present in the population—many of these populations/species exhibit year‐round breeding, and we collected pregnant females and observed/collected young juveniles in all cases. Second, for a subset of the cannibalism surveys in The Bahamas, we leveraged well‐studied systems to test for the expected differences between populations that vary in levels of resource competition (prediction 1b), predation (prediction 3), and dispersal (prediction 4). Third, we performed an outdoor mesocosm experiment to directly test the roles of resource competition (prediction 1b) and predation risk (prediction 3) on the prevalence of cannibalism in *G*. *affinis*. Further details of each study component are provided below.

In our wild‐caught fish surveys, we further measured the body size (standard length, SL) of the cannibal (in all cases) and victim (whenever degree of digestion allowed) to assess the stage of victims (e.g., newborns vs. older juveniles), the relationship between cannibal and victim body sizes, and an estimated minimum body size required to cannibalize young conspecifics in the wild (based on minimum cannibal‐victim body size ratio and estimated size of newborns from prior research). We then compared this estimated cannibal body size threshold to typical adult male and female body sizes to determine whether differences in cannibalism prevalence between species or sexes might be explained by differences in body size.

### 2.1 *Gambusia affinis* in native and invasive range

We collected 410 adult Western mosquitofish (*G*. *affinis*; Figure 1) from three populations within their native range (Oklahoma, USA) and two populations from Hawaii where they were introduced in 1905 and are highly invasive (Table S1). For the native populations, we assessed cannibalism by visually inspecting each stomach for the presence of cannibalism during dissections for life‐history analyses (part of a separate study: Riesch et al., 2016). Specifically, we dissected each fish, removed reproductive tissues, and examined the gut under a stereo microscope for the presence of a conspecific that had been eaten. For the invasive populations, we assessed cannibalism by capturing a digital x‐ray image of each fish in the lateral perspective using a custom‐built digital x‐ray unit comprising a micro‐focus x‐ray source (Hamamatsu L6731‐01) and a digital x‐ray detector (PaxScan 2520E) housed in a lead‐shielded cabinet (e.g., see Beckmann et al., 2015; Langerhans et al., 2021). We inspected each image for the presence/absence of fish within the guts: consumed fish were visible in the x‐ray images due to their dense otoliths, vertebrae, and skulls (body outline also often visible). Previous work demonstrated the feasibility of this technique—using the same x‐ray machine employed here—for the detection of vertebrate prey, especially small fish, in poeciliid guts (Beckmann et al., 2015). In all cases, the occurrence of fish in guts identified via x‐ray imaging was confirmed as cannibalism by dissection and inspection of gut contents. As a conservative measure, we further dissected and examined the gut contents for any case in which the x‐ray image indicated the presence of a dense prey that did not appear to be a fish (e.g., lacked vertebrae, shape or density not consistent with fish) but showed a somewhat similar signature (e.g., shrimp, large amphipod). We refer to these as “suggestive” x‐ray signatures. None of these cases uncovered cannibalism. For *G*. *affinis*, we dissected two fish from Hawaii with suggestive x‐ray signatures.

### 2.2 Gambusia eurystoma

*Gambusia eurystoma* is endemic to the Baños del Azufre (a toxic, hydrogen‐sulfide spring complex) in Tabasco/Chiapas in southern Mexico (Miller, 1975; Tobler et al., 2008). We collected 89 adults (Figure 1) from this locality (Table S1) and recorded the presence of cannibalism during life‐history dissections as described above (part of several separate studies: Riesch et al., 2010, 2014, 2016).

### 2.3 Gambusia geiseri

We collected 169 adult largespring gambusia (*G*. *geiseri*) from the source spring and river of the San Marcos River in Texas, USA (Table S1), and employed the x‐ray method described above to assess cannibalism. We dissected and visually examined the gut contents of one fish from each population with suggestive x‐ray signatures.

### 2.4 *Gambusia holbrooki* in native and invasive range

We collected adult Eastern mosquitofish (*G*. *holbrooki*; Figure 1) from 17 populations across their native range in North America, spanning > 14º latitude along the eastern coast of the United States (*N* = 1285, Table S2; see Riesch et al., 2014; Riesch et al., 2016, Riesch et al., 2018). These localities included lakes, ponds, ditches, springs, and toxic sulfur springs. We additionally examined adult *G*. *holbrooki* from 10 populations (lakes, ponds, canals, and ditches) from their invasive ranges in The Bahamas, Italy, and Spain (*N* = 275, Table S2). It is unknown when *G*. *holbrooki* was introduced to The Bahamas, but it was introduced to Spain in 1921 from North Carolina, USA, and transferred to Italy in 1922 (Artom, 1924; Krumholz, 1948; Nájera Angulo, 1944). With one exception, the native‐range collections were examined using life‐history dissections described above; the European collections were dissected as part of a diet study using gut‐content analysis (Pirroni et al., 2021); the Bahamian collection and the native‐range collection from Big Pine Key, FL were x‐rayed using the method described above. We dissected and visually inspected the gut contents of one fish with a suggestive x‐ray signature from the Bahamian collection.

### 2.5 *Gambusia hubbsi*, *G*. *manni*, and *G*. sp. from The Bahamas

Three endemic species of *Gambusia*, which form a monophyletic clade of closely related species, inhabit the islands of The Bahama Archipelago. While *G*. *manni* inhabits eastern and southern islands of the Great Bahama Bank, *G*. *hubbsi* (Figure 1) inhabits north‐western islands of the Great Bahama Bank, and a so‐far unnamed species, *Gambusia sp*., inhabits the islands of the Little Bahama Bank (e.g., Giery et al., 2015; Heinen‐Kay et al., 2014; Riesch et al., 2015). We collected adult individuals of all three species from a large number of disparate localities across eight islands (2–4 per species) in The Bahamas (Table 1, Table S3). These collections comprise three major habitat types: (1) tidal creeks, (2) inland blue holes, and (3) ponds. Using cannibalism estimates across all three types of habitats, we examined the overall prevalence of cannibalism and whether more dispersal‐limited environments exhibited lower rates of cannibalism (see below). For a subset of these populations (46 tidal creeks, 21 blue holes), we have detailed information regarding population density, resource availability, and predation risk (e.g., Heinen et al., 2013; Heinen‐Kay et al., 2014; Langerhans, 2018; Langerhans et al., 2007; Riesch et al., 2015, 2020), and use these sites for tests of associations between ecological drivers and cannibalism (see below).

**TABLE 1:** Sample locations, year of sampling, sample sizes, occurrence of cannibalism, and method of assessment (XR: x‐ray imaging, LH: life‐history dissections, DI: diet analysis of stomach contents) for adult *Gambusia hubbsi* males and females from the 21 focal blue holes on Andros Island, The Bahamas. If no number precedes the method of assessment, then all specimens were examined using that method (multiple methods could be used per specimen)

Predation regime	Population	Year	Latitude	Longitude	Cannibalism by males	Cannibalism by females	Prevalence of cannibalism (%)	Method(s)
Low	Archie's	2002	24.90137	−77.93621	0/53	3/89	2.1	XR
		2004			0/11	0/8	0.0	XR
		2012			0/21	0/46	0.0	XR
	Douglas Christopher	2010	24.23947	−77.67702	1/30	0/30	1.7	XR
	East Twin	2006	24.75154	−78.00581	0/10	–	0.0	XR
		2010			–	0/8	0.0	XR
		2011			0/32	1/32	1.6	XR, 40 DI
		2012			1/22	–	4.5	XR, 22 LH
		2013			–	0/16	0.0	XR
	Gabbler	2002	24.61815	−77.76305	0/7	1/84	1.1	XR
		2004			0/13	–	0.0	XR
	Gollum	2004	24.80059	−78.01686	0/15	4/46	6.6	XR
		2009			0/18	0/16	0.0	XR
		2011			0/30	0/33	0.0	XR
	Hubcap	2004	24.77580	−77.85768	0/13	1/32	2.2	XR
		2011			0/31	0/39	0.0	XR, 41 DI
		2012			0/15	–	0.0	XR, 15 LH
	Ken's	2004	24.81985	−78.07851	1/10	0/11	4.8	XR
		2011			0/32	0/32	0.0	XR, 37 DI
	Little Frenchman	2004	24.50700	−77.72220	0/46	0/61	0.0	XR
	Pigskin	2006	24.68759	−78.03084	0/10	1/7	5.9	XR
		2011			0/31	2/33	3.1	XR, 32 DI
	Rainbow	2002	24.78501	−77.85995	0/7	0/46	0.0	XR
		2004			0/46	0/88	0.0	XR
		2005			0/36	0/28	0.0	XR
		2011			0/25	1/35	1.7	XR, 62 DI
	Voy's	2011	24.88363	−77.96945	0/25	7/39	10.9	XR, 44 DI
High	Cousteau's	2002	24.77639	−77.91598	0/5	0/6	0.0	XR
		2004			0/32	0/8	0.0	XR
		2005			0/54	0/39	0.0	XR
		2011			0/22	0/18	0.0	XR, 10 DI
		2012			0/32	–	0.0	XR, 32 LH
	Gibson	2004	24.77381	−77.90460	0/20	0/22	0.0	XR
		2011			0/20	0/19	0.0	XR
	Goby Lake	2004	24.82850	−77.92310	0/2	0/6	0.0	XR
		2011			0/13	0/11	0.0	XR
	Hard Mile	2004	24.77590	−78.03724	0/20	0/16	0.0	XR
		2011			0/10	0/10	0.0	XR
	Murky Brown	2004	24.78703	−77.91145	0/20	0/21	0.0	XR
		2011			0/10	0/5	0.0	XR
	Rivean's	2004	24.50562	−77.74843	0/24	0/58	0.0	XR
		2011			0/31	0/10	0.0	XR
	Runway	2006	24.72846	−77.98114	0/30	0/25	0.0	XR
		2011			0/10	0/11	0.0	XR
	Shawn's	2004	24.73281	−77.86893	0/22	0/10	0.0	XR
		2011			0/25	0/29	0.0	XR, 14 DI
	Stalactite	2004	24.78543	−78.01679	0/6	0/15	0.0	XR
		2005			0/19	0/31	0.0	XR
		2011			0/31	0/28	0.0	XR
		2012			0/21	–	0.0	XR, 21 LH
	West Twin	2006	24.75265	−78.00855	0/10	0/10	0.0	XR, 29 DI
		2011			0/32	0/36	0.0	XR
		2012			0/22	–	0.0	XR, 22 LH
		2013			–	0/18	0.0	XR
		Total			3/1132	21/1321	0.98	

In total, we examined 8,081 adult Bahamian mosquitofish. Cannibalism was assessed using x‐ray imaging as described above for 7,586 fish, while we conducted a direct examination of gut contents during diet analyses for 495 additional fish (all from Abaco Island; see Araújo et al., 2014; Langerhans et al., 2021). We further dissected and visually examined the guts of 1,680 of the x‐rayed specimens as part of life‐history and/or diet analyses (716 from tidal creeks, 882 from blue holes, 82 from ponds; e.g., see Riesch et al., 2013; Riesch et al., 2015; Riesch et al., 2016, Riesch et al., 2020). During the latter dissections, we never encountered discrepancies between x‐ray scored cases of cannibalism and visual observations. We additionally dissected eight specimens with suggestive x‐ray signatures.

Because poeciliid fish can discriminate kin, we did not expect to find reduced cannibalism in more dispersal‐limited populations, as would be predicted if the risk of consuming kin were greater in these environments (e.g., Boots et al., 2021). To test this, we made two comparisons: we tested (1) whether tidal creeks with severe hydrological restriction from the ocean due to human‐induced fragmentation (see details below) had lower rates of cannibalism than unfragmented tidal creeks, and (2) whether blue holes, which are geographically isolated and known to exhibit minimal gene flow with other populations (e.g., Langerhans et al., 2007; Riesch et al., 2013; Schug et al., 1998), had lower cannibalism prevalence than other habitat types. The premise being that both of these habitat types (fragmented tidal creeks, blue holes) should exhibit much lower levels of dispersal than the counterparts they were compared to, and thus have higher frequencies of encountering kin.

To test whether cannibalism prevalence was higher in populations with higher resource competition (estimated with population density and resource availability) or lower predation risk (estimated with presence/density of predatory fish), we utilized two separate systems with considerable prior research: tidal creeks and inland blue holes. First, Bahamian tidal creeks are shallow, tidally influenced estuaries typically having a relatively narrow creek mouth that broadens landward. Water flux in these systems primarily arises from tidal exchange (freshwater input only provided via rainfall and aquifer percolation), so salinities in unfragmented systems are typically around 35 ppt and the biotic communities comprise marine taxa (Araújo et al., 2014; Layman et al., 2004; Riesch et al., 2015; Valentine‐Rose et al., 2007a, 2007b). Widespread fragmentation of Bahamian tidal creeks throughout The Bahamas, primarily caused by road construction (mostly during the 1960s and 1970s), has resulted in severe restriction of hydrological connectivity with the ocean. This pervasive ecosystem fragmentation has caused strong and consistent ecological changes in tidal creeks—for example, reduced tidal exchange, reduced species diversity, increased density of *Gambusia*, decreased density (or extirpation) of piscivorous fish (e.g., Layman et al., 2004; Valentine‐Rose et al., 2007a, 2007b)—and led to a number of phenotypic shifts in Bahamian mosquitofish (e.g., Giery et al., 2015; Heinen‐Kay et al., 2014; Jenkins et al., 2021; Riesch et al., 2015), including dietary changes (Araújo et al., 2014; Langerhans et al., 2021). Prior work has characterized many of these tidal creeks regarding the population density of *Gambusia* and the density of predatory fish using visual surveys, and we aimed to use general linear models to test for associations between these variables and cannibalism in this study (*N* = 3,173 specimens from 24 fragmented and 22 unfragmented tidal creeks). However, owing to the extreme rarity of cannibalism in these environments (see Results), we simply evaluated the occurrence of cannibalism in fragmented and unfragmented tidal creeks.

Second, inland blue holes are water‐filled, vertical caves that are characterized by a freshwater lens (or brackish on some islands) overlying denser saltwater (Björnerås et al., 2020; Mylroie et al., 1995). Blue holes are common in The Bahamas, and during the past ~15,000 years (Fairbanks, 1989), Bahamian mosquitofish have colonized a large number of inland blue holes throughout these islands. In the central‐northern areas of Andros Island, *G*. *hubbsi* have subsequently undergone adaptive diversification in a large number of traits (reviewed in Langerhans, 2018) and evolved varying levels of reproductive isolation among populations (e.g., Langerhans et al., 2007; Langerhans & Makowicz, 2013). This adaptive radiation apparently stems from strong and temporally consistent variation among blue holes in predation risk and resource competition, while other environmental variables show little variation, or no covariation with these primary drivers. Specifically, in some blue holes *G*. *hubbsi* experience a relatively predator‐free environment devoid of any piscivorous fish, and consequently exhibit high population densities with elevated competition for food resources. In other blue holes, *G*. *hubbsi* are heavily preyed upon by the much larger bigmouth sleeper (*Gobiomorus dormitor*) and have much lower population densities (e.g., Heinen et al., 2013; Langerhans et al., 2007; Martin et al., 2015; Riesch et al., 2020). Independently, these blue holes also differ consistently in the availability of key resources (i.e., zooplankton density; Heinen et al., 2013; Hulthén et al., 2021), which has influenced the evolution of fin color and life histories in *G*. *hubbsi* (Hulthén et al., 2021; Martin et al., 2014; Riesch et al., 2020). Because other abiotic environmental variables do not covary with predator presence or resource availability (e.g., Björnerås et al., 2020; Heinen et al., 2013; Langerhans et al., 2007; Riesch et al., 2013), this system provides a remarkable opportunity to test for the role of predation risk and resource competition on the prevalence of cannibalism in the wild.

Using 21 blue holes on Andros Island with considerable prior research (11 low‐predation and 10 high‐predation), we tested for increased occurrence of cannibalism in low‐predation compared to high‐predation populations (*N* = 2453, Table 1) using a two‐sample binomial proportions test. For each site, we calculated the overall proportion of fish with cannibalized individuals in their guts (pooled across sexes and years).

To test whether resource competition, or some other feature associated with conspecific population density, might explain variation in cannibalism prevalence, we examined variation in *G*. *hubbsi* resources and density. We have previously measured zooplankton density for 18 of these populations, and repeatedly measured population density for 17 of these populations (e.g., Heinen et al., 2013; Hulthén et al., 2021; Martin et al., 2014; Riesch et al., 2020). To eliminate any potential confounding role of predation risk, we restricted our analysis to low‐predation blue holes (no predatory fish present; 8 populations had population density and zooplankton density data). For statistical analysis, we arc‐sin square‐root transformed proportional cannibalism for use as a dependent variable that met assumptions of linear models. Note that results were very similar, and qualitatively unchanged, if we instead used a generalized linear model with a binomial error distribution and logit link function. To test the prediction that cannibalism prevalence will increase under more intense resource competition, we conducted a multiple regression using arc‐sin square‐root transformed proportional cannibalism as the dependent variable and log_10_‐transformed population density and log_10_‐transformed zooplankton density as the independent variables. We predicted that cannibalism would increase in prevalence with higher population densities and lower zooplankton density. To rule out the possibility that encounter rates with juveniles might explain any of these findings, we also tested for a correlation between arc‐sine square‐root transformed proportional cannibalism and arc‐sine square‐root transformed proportion of juveniles in the populations based on previous studies (prior work characterized overall population density and the proportion of juveniles, repeatedly, in many blue holes: e.g., Heinen et al., 2013; Riesch et al., 2020). Multicollinearity was low (VIF = 1.0) and data met assumptions of normality of residuals.

### 2.6 Gambusia melapleura

We collected 47 adult *G*. *melapleura* from their type locality stream in Bluefield, Jamaica (Table S1), and used the x‐ray method described above to examine cannibalism.

### 2.7 Gambusia panuco

We collected 58 adult *G*. *panuco* from a stream in Tamaulipas, Mexico (Table S1), and again used the x‐ray method to examine cannibalism.

### 2.8 Gambusia puncticulata

We collected *G*. *puncticulata* from the Cayman Islands and Jamaica. There is some disagreement regarding the taxonomic status of these *Gambusia*: that is, whether the forms represent endemic species (*G*. *caymanensis* in the Cayman Islands, *G*. *oligosticta* in Jamaica), or are synonymous with *G*. *puncticulata* in Cuba (e.g., Fink, 1971; Greenfield & Wildrick, 1984; Rauchenberger, 1989; Rivas, 1963). Recent molecular work suggests the forms represent recent colonizations from Cuba and lack reciprocal monophyly (<200,000 years ago; Lydeard et al., 1995; R.B. Langerhans, M.E. Gifford, O. Domínguez‐Domínguez & I. Doadrio unpubl. data). Thus, we refer to these taxa here as *G*. *puncticulata*.

We collected 546 adult *G*. *puncticulata* from nine populations across the three Cayman Islands (Table S1; see Langerhans & Makowicz, 2009). We used the x‐ray imaging method described above for all fish, and additionally employed the same method of visual inspection of stomachs during life‐history collections as described above for 155 of these individuals. Owing to the lack of prior diet studies in this species in the Cayman Islands, we examined the gut contents of 69 adults (35 females, 34 males) from five populations to confirm similar diets to other *Gambusia* species examined here. Based on these observations, it appears *G*. *puncticulata* in the Cayman Islands regularly consumes insects and crustaceans, like other mosquitofishes, but also contains a larger amount of algae/plant material and detritus in their diet in some localities than most other *Gambusia* species. We collected 82 adult *G*. *puncticulata* from two populations in Jamaica (Table S1) and employed the x‐ray method described above to assess cannibalism.

### 2.9 Gambusia quadruncus

We examined 56 adult llanos mosquitofish (*G*. *quadruncus*) from four populations in Mexico (Table S1; see Langerhans et al., 2012), and again employed our x‐ray method for the assessment of cannibalism.

### 2.10 Gambusia rhizophorae

Using the same x‐ray method, we examined 68 adult mangrove gambusia (*G*. *rhizophorae*; Figure 1) collected from two populations in Florida (Table S1) for cases of cannibalism.

### 2.11 Gambusia sexradiata

We collected 125 adult *G*. *sexradiata* (Figure 1) from two populations in Tabasco, Mexico (Table S1) and recorded the presence of cannibalism during life‐history dissections as described above.

### 2.12 Gambusia vittata

We collected 45 adult *G*. *vittata* from one population in Tamaulipas, Mexico (Table S1), and used the x‐ray method described above to examine cannibalism.

### 2.13 Gambusia wrayi

We collected 58 adult *G*. *wrayi* from two populations in Jamaica (Table S1) and used the x‐ray method described above to examine cannibalism. We dissected and visually examined the gut contents of one fish with a suggestive x‐ray signature.

### 2.14 Heterophallus milleri

The Grijalva gambusia (*H*. *milleri*) and two other species (*H*. *echeagarayi*, *H*. *rachovii*) comprise a sister clade to the rest of the genus *Gambusia* (Hrbek et al., 2007; Miller, 2005; Radda, 1987; R. B. Langerhans et al. unpubl. data), and disagreement exists regarding whether these species belong to the *Gambusia* genus or their own genus *Heterophallus*. Regardless, *H*. *milleri* exhibits a similar natural history, diet, and life history to other *Gambusia* species (Riesch et al., 2011). We collected 75 adult *H*. *milleri* from one population in Tabasco, Mexico (Table S1) and recorded the presence of cannibalism during life‐history dissections as described above.

### 2.15 *Poecilia reticulata* in native and invasive range

We examined 292 adult Trinidadian guppies (*P*. *reticulata*) from 12 populations in their native range on Trinidad and 185 adult guppies from three populations in O'ahu, Hawaii where they were introduced approximately 100 years ago (Brock, 1960; Rosenthal et al., 2021) (Table S1; see Santi et al., 2021). These localities include a natural asphalt lake (Pitch Lake), several oil‐polluted sites, and unpolluted ponds, ditches, and streams. The native‐range specimens were dissected as part of life‐history analyses (Santi et al., 2021) and gut‐content analysis (D. Lucion & R. Riesch, unpubl. data), while we assessed cannibalism in the invasive‐range specimens using the x‐ray method described above. We dissected and visually examined the guts of three fish from Hawaii with suggestive x‐ray signatures.

### 2.16 Mesocosm experiment with *Gambusia affinis*

To examine the roles of intraspecific resource competition and predation risk (Predictions 1b and 3) on cannibalism in adult *G*. *affinis*, we performed a 7‐day, outdoor mesocosm experiment with a 2 × 3 factorial randomized block design that directly manipulated *G*. *affinis* density (low vs. high) and predation risk (no‐predator, caged lethal‐predator present, uncaged lethal‐predator present), while holding the initial resource levels and other environmental settings relatively constant. We constructed 36 mesocosms to simulate natural conditions and performed the experiment in six blocks during summer 2008 (19 June – 24 August) in an open field at the University of Oklahoma Biological Station (Kingston, OK, USA). Each block used six mesocosms, one for each of the six treatment combinations. Mesocosms comprised 183 cm diameter × 61 cm high polyethylene tanks, filled to approximately 40 cm depth (1050 L), with artificial plants around the perimeter (12 evenly spaced and vertically oriented 55‐cm long, dark green polypropylene ropes glued to the bottom and frayed at the top 20 cm), large benthic structure on the bottom (4 evenly spaced, 19‐cm diameter plastic plates glued to the bottom 40 cm from the edge with a 120‐cm length bundle of the polypropylene rope glued to its center), and small benthic structure along the bottom (3 10‐cm long 1.25‐cm ∅ pvc pipes with 2 7.5‐cm long frayed polypropylene ropes coming from one end situated around each plate). Mesocosms were covered with shade cloth during the experiment to exclude avian predators and minimize amphibian/insect colonization.

All animals used in the experiment were collected in the nearby reservoir of the Red River, Lake Texoma (approximately 300 m from mesocosm array). Prior to experimentation, *G*. *affinis* individuals were held in an outdoor 2,400‐L stock tank for 2–4 days, predatory fish (largemouth bass, *Micropterus salmoides*) were held in two separate 2400‐L stock tanks for several weeks, and aquatic invertebrate prey were held in two separate 2400‐L stock tanks for several days (collected using a 80‐µm plankton net and a LaMotte D net). All stock tanks had mechanical filtration and ample aeration, and we fed animals twice per day while in the stock tanks (Tetra goldfish flakes for *G*. *affinis*, Hikari pellet food for largemouth bass, crushed Tetra goldfish flakes and Zeigler spirulina flakes for aquatic invertebrates).

The low‐density treatment had 10 *G*. *affinis* (8 females, 2 males) and the high‐density treatment had 30 *G*. *affinis* (24 females, 6 males). We used more females than males to approximate the local natural sex ratio and because females tend to show higher rates of cannibalism than males. Moreover, we did not introduce juvenile *G*. *affinis* into mesocosms, but rather allowed pregnant females to deliver newborn offspring during the experiment, which provided potential juvenile victims. Most females were visibly pregnant prior to experimentation, and delivery of offspring occurred in most, if not all, mesocosms (see Results). Each *G*. *affinis* was individually marked using Visible Implant Elastomer (VIE) tags (Northwest Marine Technology, Inc.) with two small marks of three possible colors (red, yellow, blue), and allowed to recover for 3–4 days before experimentation. All *G*. *affinis* were photographed for measurement of standard length (SL, using the program tpsDig2 ver. 2.14, Rohlf, 2009) and weighed to the nearest 0.0001 g the day before they were introduced into mesocosms; all surviving *G*. *affinis* were collected and re‐weighed at the conclusion of the experiment to measure their change in weight. These methods for body‐size estimation have been shown to have very low measurement error (i.e., very high repeatability measured as intraclass correlation coefficient; Langerhans et al., 2021). To estimate average weight change per replicate, we first subtracted the initial weight from the final weight of each individual, and then regressed this against log_10_‐transformed SL and saved the residuals. This procedure adjusted for the fact that smaller fish showed a larger weight change, resulting in a size‐corrected estimate of weight change. We then calculated the average value for each replicate. We assumed a lower average change in weight per mesocosm during the experiment at least partially reflected reduced growth rates/condition potentially related to resource competition. We further assumed that unrecovered *G*. *affinis* reflected mortality that occurred during the experiment.

To accommodate the predator treatments, each mesocosm had a nylon mesh cage in the center (25 cm × 25 cm × 46 cm). The cage was empty in the no‐predator and lethal‐predator treatments but contained a single largemouth bass in the caged‐predator treatment. A single largemouth bass was free to roam the mesocosm in the lethal‐predator treatment. Thus, there were no visual or chemical cues of predatory fish in the no‐predator treatment, visual and chemical cues of a predator that could not actually consume *G*. *affinis* in the caged‐predator treatment, and the potentially lethal presence of a predatory fish, along with its visual and chemical cues, in the lethal‐predator treatment. The inclusion of the caged‐predator treatment allowed us to evaluate the indirect role of predators on cannibalism prevalence through only the perceived predation risk and not any subsequent reduction in density. Meanwhile, the inclusion of the lethal‐predator treatment permitted us to determine the combined direct and indirect roles of predators on cannibalism prevalence by allowing predators to not only induce altered prey behaviors but also reduce prey density. We used 20 individual largemouth bass in the experiment (mean body size ± std. err. = 10.5 ± 0.19 cm SL).

Each block lasted a total of 12 days, with a single largemouth bass added to the predator treatments on Day 4 and *G*. *affinis* added on Day 5 (predators were temporarily removed for 2 hrs to allow acclimation of *G*. *affinis*; the predator cage was manipulated at this time in the no‐predator treatment so that all tanks experienced similar disturbance). During each block, we randomly assigned treatments to tanks, filled six mesocosms with municipal water (Day 1), left them uncovered for 24 h, added 37 L of water from Lake Texoma and covered them with shade cloth (Day 2), recorded abiotic water conditions several days at 10:00 (Days 3–5 and Day 12; temperature, pH, dissolved oxygen, conductivity, salinity), added aquatic invertebrates and crushed Tetra goldfish flakes / Zeigler spirulina flakes (Day 3), and ended the experiment on Day 12. To standardize the amount of aquatic invertebrates added to each replicate within each block, we performed the following procedure: (1) conducted 5 sweeps with the plankton net in the open water of each of the two invertebrate stock tanks and pooled these collections into an 18L container, (2) conducted 2 sweeps along the bottom of each stock tank with the D net and pooled these collections into a separate 18L container, (3) collected 120 *Physa* snails and 120 amphipods, and (4) added to each of the six mesocosms 2L of stirred water from each 18L container, 20 *Physa* snails, and 20 amphipods. On Day 12 (after *G*. *affinis* had been in each tank for seven days), we removed largemouth bass and *G*. *affinis* at approximately 10:30, and subsequently took a sample of pelagic and benthic resources within each mesocosm by conducting 5 sweeps of the plankton net in the open water and 5 sweeps of the D net along the bottom. We preserved, identified, and counted all possible prey from these samples to quantify resource availability at the end of the experiment. We euthanized and preserved each *G*. *affinis* for gut‐content analysis, where we identified and counted all diet items in the guts. As we center on cannibalism in this study, we only present summary information here for resource availability and diet—future studies will present more detailed analyses.

We conducted analysis of variance (ANOVA) to test for variation among treatments in abiotic variables, resource availability (total number of prey items, log_10_‐transformed), juvenile density (number of juveniles collected in resource sampling), and cannibalism prevalence. We calculated the prevalence of cannibalism as the number of observed instances of cannibalism per replicate divided by the number of surviving adults recovered at the end of the experiment within that mesocosm that had prey items observed in their stomach (arc‐sin square‐root transformed). To adjust for any variation among blocks, all dependent variables were standardized to a mean of zero and standard deviation of one within each block. Our ANOVAs included the density treatment, predation treatment, and their interaction as independent variables. Note that results for cannibalism were very similar, and qualitatively unchanged, if we instead used a generalized linear model with a binomial error distribution and random effect of Block, or if we calculated cannibalism relative to the total number of surviving adults irrespective of whether their stomachs had food items.

We further used Pearson correlation tests to more directly examine a number of hypothesized associations. Specifically, to test the hypothesis that higher density resulted in more intense intraspecific resource competition, we tested for negative associations between final adult density and both resource availability and average weight change. We tested whether juvenile *G*. *affinis* density simply reflected higher adult densities by looking for a positive correlation between the two variables. We tested for a positive association between cannibalism prevalence and both final adult density and juvenile density. To provide more direct tests of the effects of resource competition on cannibalism, we tested for a negative association between cannibalism prevalence and both resource availability and average weight change. Residuals were approximately normal.

## 3 RESULTS

### 3.1 Broad‐scale surveys of Cannibalism

#### 3.1.1 Rarity

**FIGURE 2:**
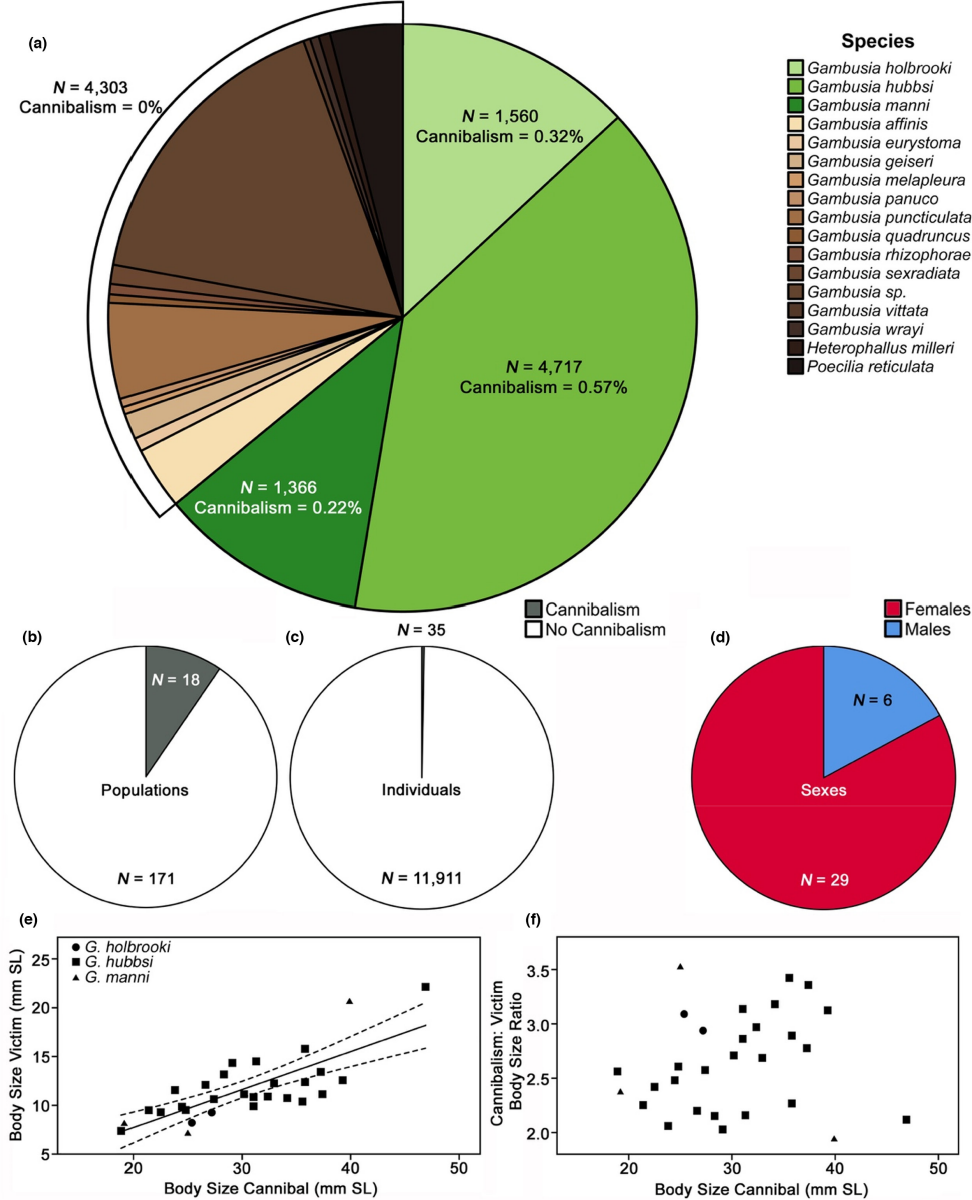
Proportional to total sample size, (a) the species for which we found evidence for cannibalism (green) relative to species for which we found no evidence for cannibalism (brown), and (c) the number of individuals for which we found evidence for cannibalism (gray) relative to the number of individuals for which we found no evidence for cannibalism (white). Proportional to the total number of populations, (b) the number of populations for which we found evidence for cannibalism (gray) relative to the number of populations for which we found no evidence for cannibalism (white). Proportional to the total number of identified cannibals, (d) the number of female (red) to male (blue) cannibals. The significant relationship (e) between the body size of the victim and the body size of the cannibal with best‐fit line and 95% confidence interval and the non‐significant pattern (f) between the cannibal‐to‐victim body size ratio and the body size of the cannibal

**FIGURE 3:**
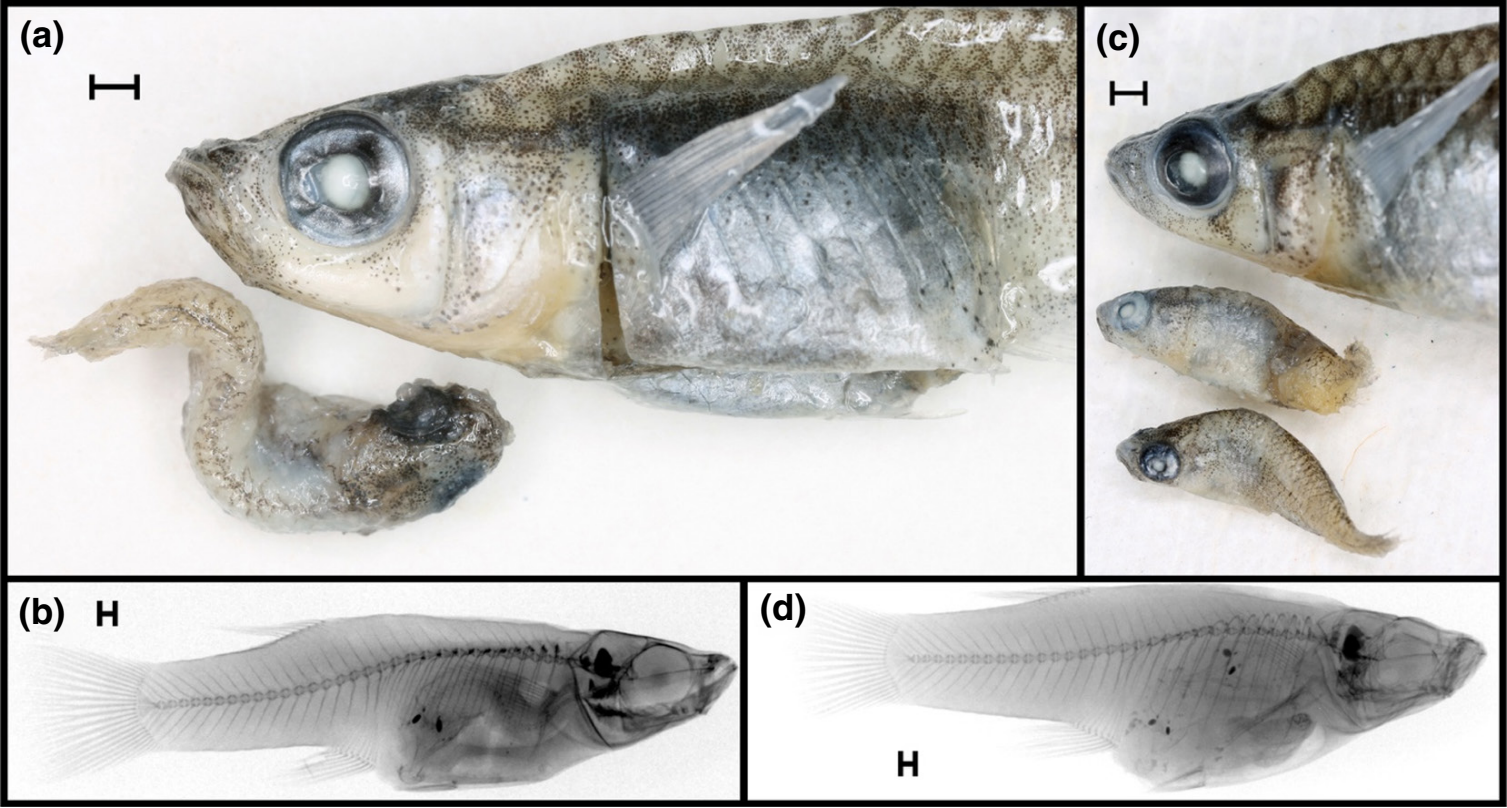
Representative, paired photographs and x‐ray images of (a, b) a cannibalistic *Gambusia hubbsi* female from Rainbow and (c, d) Pigskin blue holes. Photographs (a, c) show the anterior body region of the cannibalistic female along with the cannibalized victim(s) removed from her gut during dissections; note how the spine and otoliths of the cannibalized victims are clearly visible in the associated x‐rays (b, d). Scale bars represent 1 mm

Overall, cannibalism in *Gambusia* spp. and guppies in the wild was quite rare (Figure 2). Across all 11,946 wild‐caught adult mosquitofish and guppies, we only observed cannibalism in 35 individuals (0.3% occurrence; Figure 2b, c). We never observed cannibalism in 14 of the 17 species examined (0 of 4290 individuals; Figure 2a, Tables S1‐S3). For the three species where cannibalism was observed, overall occurrence was rare: *G*. *holbrooki*: 0.3%, *G*. *hubbsi*: 0.6%, *G*. *manni*: 0.2% (Figure 2a). Rarity of cannibalism was not explained by a general lack of detection of prey within guts, as prey items were readily observable in the majority of specimens via x‐images or visual inspection of guts. For instance, while cannibalism in tidal creeks was very rare, with only two observed cases out of 3,513 fish in The Bahamas (0.06%; both observed in x‐ray images), gut‐content analyses in these populations uncovered 4,837 prey items in 542 specimens (71% of individuals had food items present in their guts). Within *G*. *holbrooki*, cannibalism was only observed in the native range, not in the invasive range. Most of the observed cases of cannibalism occurred in *G*. *hubbsi* populations inhabiting inland blue holes that lack predatory fish (Table 1; see below). Even within populations where cannibalism occurred, it was generally rare (average of 3.6% across all such collections); only twice did the prevalence of occurrence reach approximately 11% within populations (Table 1, Table S3). Of the 35 cannibalistic individuals, all but two had consumed a single conspecific individual: two female *G*. *hubbsi* had consumed two conspecifics (one in Archie's blue hole and one in Pigskin blue hole). Figure 3 illustrates two examples of cannibalistic females.

#### 3.1.2 Female bias and body size

Cannibalism was more common in females than in males. Overall, we found 29 of 7,342 females (0.4%) and 6 of 4,591 males (0.1%) exhibited cannibalism (Figure 2d). If we exclude species where no cannibalism was observed, these numbers remain relatively similar: 29 of 4,567 females (0.6%), 6 of 3,076 males (0.2%). Cannibalistic individuals spanned a large range of body size: 17.5–46.9 mm SL (mean = 29.0 mm). Cannibalistic females ranged from 21.4 to 46.9 mm SL (mean = 30.5 mm), while males ranged from 17.5 to 28.4 mm SL (mean = 21.9 mm).

All cannibalized victims were juveniles except for two mature males (consumed by *G*. *hubbsi* in London Pond on Andros Island and by *G*. *manni* in Clear Pond on San Salvador Island). We estimated SL for 29 of the cannibalized victims, as the remaining 8 victims were too digested for measurement of body size. Cannibalized victims ranged in size from 7.1 to 22.1 mm SL (mean = 11.7 mm). The largest juvenile consumed was 15.8 mm SL, while the consumed males were 20.6 and 22.1 mm SL. Exclusively within Androsian blue holes, where much of the observed cannibalism occurred, *G*. *hubbsi* victims ranged from 9.3 to 15.8 mm SL (mean = 11.6 mm SL). Larger individuals tended to consume larger victims (*r* = 0.76, *p* < 0.0001; Figure 2e). The average cannibal‐victim size ratio was 2.65 (range 1.94–3.52), and this ratio was unrelated to the body size of the cannibal (*r* = 0.13, *p* = .50; Figure 2f).

#### 3.1.3 Association with dispersal limitation

We found no support for the notion that more dispersal‐limited populations would exhibit lower cannibalism prevalence since these individuals might consume their own offspring (or other kin) at a higher likelihood. As predicted based on the presence of kin recognition in poeciliid fish, we neither observed higher cannibalism prevalence in unfragmented compared to fragmented Bahamian tidal creeks nor lower cannibalism prevalence in inland blue holes compared to other habitat types within The Bahamas. Indeed, the only two cases of cannibalism found in tidal creeks were observed within fragmented, not unfragmented tidal creeks (Table S3). Moreover, inland blue holes showed *higher* cannibalism prevalence than other habitat types (Table 1, Table S3).

### 3.2 Effects of resource competition and predation on cannibalism in Bahamian mosquitofish

Because of the extreme rarity of cannibalism in tidal creeks (1 of 46 populations), we could not test for associations between cannibalism and population density or piscivore density within these environments. While we did observe higher cannibalism prevalence in fragmented tidal creeks (1 of 24 populations) than unfragmented tidal creeks (0 of 22 populations), the occurrence of cannibalism was so rare that no conclusions can be made regarding any differences between fragmentation regimes.

**FIGURE 4:**
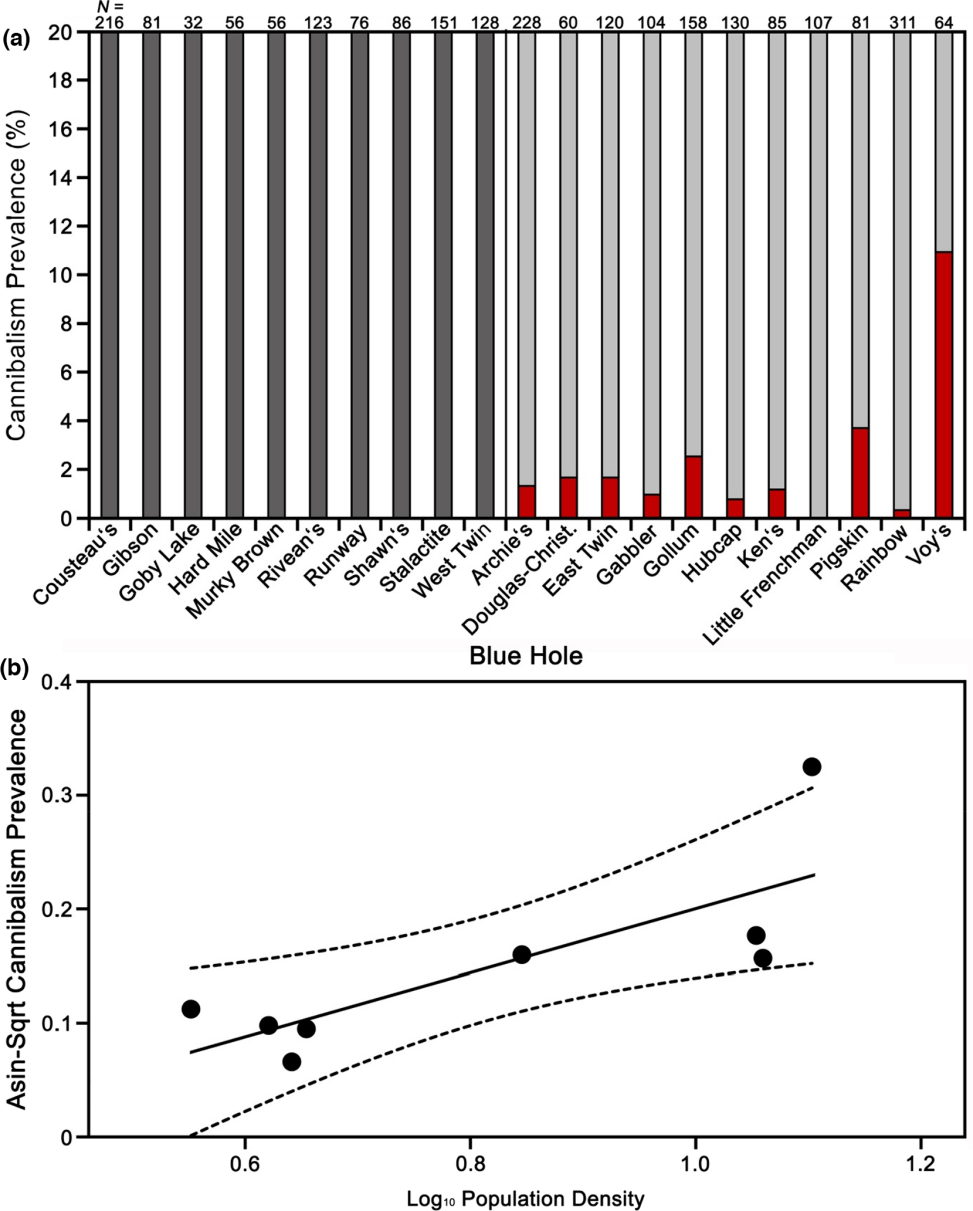
Cannibalism prevalence in (a) *Gambusia hubbsi* from blue holes in The Bahamas (from Cousteau's to West Twin are high‐predation blue holes and from Archie's to Voy's are low‐predation blue holes). Red indicates the proportion of fish that did cannibalize, and gray represents the proportion of fish that did not (dark gray: high predation; light gray: low predation); please note that for visualization purposes, y‐axes were capped at 20%. (b) Relationship between cannibalism prevalence and population density in low‐predation blue holes in The Bahamas, with best‐fit line and 95% confidence intervals

Within the 21 focal blue holes on Andros Island (Table 1), we found higher cannibalism prevalence in *G*. *hubbsi* within low‐predation blue holes (10 of 11 populations; *N* = 1,448) compared to high‐predation blue holes (0 of 10 populations; *N* = 1,005; two‐sample binomial proportions test: *z* = 4.17, *p* < .0001; Figure 4a). While *G*. *hubbsi* in low‐predation blue holes exhibited the highest prevalence of cannibalism among taxa examined in this study, it was still rare, even in those populations where it occurred (Table 1). In these blue holes, females accounted for most cannibalism (87.5%). Multiple regression conducted strictly within low‐predation populations revealed that cannibalism prevalence increased with increasing population density (*F*_2,5_ = 8.12, *p* = .0358; Figure 4b), but we found no statistical support for the negative association between cannibalism prevalence and zooplankton density (*F*_2,5_ = 0.91, *p* = .3833). The positive association between population density and cannibalism could not be explained by a correlation with the proportion of juveniles in the population, as we found no evidence for an association whether in a univariate (*r* = 0.22, *p* = .60) or multiple‐regression context (*F*_2,4_ = 0.00, *p* = 1.00).

### 3.3 Mesocosm experiment with *Gambusia affinis*

Based on the measurements made within each replicate during four separate days of the experiment within each block, we found that abiotic water conditions were relatively similar across all treatments (ANOVAs: all *p* > .05; Table S4). Samples of resource availability within mesocosms at the end of the experiment uncovered a total of 5,023 potential prey items of 30 prey categories, with the most abundant being *Daphnia* sp. (34.8% by number), chironomid larvae (22.1%), amphipods (14.1%), and cyclopoid copepods (7.7%). The high‐density treatment had much lower resource availability than the low‐density treatment (*F*_1,30_ = 9.13, *p* = .0051), while the lethal‐predator treatment had much higher resource availability than the other two predator treatments (*F*_2,30_ = 15.02, *p* < .0001), and no interaction between treatments was evident (*F*_2,30_ = 1.76, *p* = .19). The latter result demonstrated that the density treatment tended to have similar effects on resource availability within all predator treatments, even the lethal‐predator treatment where density declined during the experiment. The final adult *G*. *affinis* density was strongly negatively associated with resource availability at the end of the experiment (*r* = −0.61, *p* = .0001); higher final densities also resulted in lower average weight change in adult *G*. *affinis* (*r* = −0.41, *p* = .0169). Together, these results indicate that the foundational assumption for the experiment was met, i.e., population density affects intraspecific resource competition in semi‐natural replicates.

Of the 720 individually marked *G*. *affinis* we introduced into the 36 replicates, we recovered a total of 518 alive at the end of the experiment. One mesocosm experienced high mortality (47%) for unknown reasons (high‐density, caged‐predator treatment), and we excluded this tank from all analyses of cannibalism. No dead *G*. *affinis* were observed in any other mesocosm during the experiment. Overall, survival of *G*. *affinis* was very high in the no‐predator (99.2%) and caged predator treatments (97.9%), regardless of density, but survival was greatly reduced in the lethal predator treatment (27.2%) (Table S4). All non‐lethal replicates experienced survivorship of ≥90%, while no lethal replicate exhibited survivorship greater than 50% (within the lethal‐predator treatment, the high‐density replicates still typically had more than twice as many survivors as the low‐density replicates). Thus, virtually all unrecovered fish in the lethal treatments likely reflected predation by largemouth bass. Because one replicate (low‐density, lethal‐predator treatment) experienced 100% mortality, it was not included in analyses below since cannibalism could not be assessed.

Most of the replicates (at least 29) had newborn *G*. *affinis* delivered during the experiment (i.e., juveniles visually observed, collected in resource sampling, or found in guts of adults). A total of 76 *G*. *affinis* juveniles were recovered in the resource‐availability sampling at the end of the experiment. We did not collect more juveniles in replicates with higher final adult densities (*r* = −0.05, *p* = .75), nor was this estimated juvenile density associated with any experimental treatments (ANOVA: all *p* ≥ .70). All results of cannibalism analyses were qualitatively similar if we excluded replicates without evidence for the presence of juvenile *G*. *affinis*.

Diet examination of the 518 surviving adult *G*. *affinis* uncovered 2,861 prey items belonging to 44 prey categories within 446 fish (52 individuals had empty guts). The most common prey consumed in mesocosms were adult insects (33.2% of diet items; 46.1% occurrence), insect larvae/pupae (22.7% of diet items; 45.0% occurrence), and zooplankton (32.3% of diet items; 16.6% occurrence). Most of the remaining prey included amphipods (2.0% of diet items; 6.4% occurrence), gastropods (1.7% of diet items; 5.0% occurrence), and algae/phytoplankton/plant matter (6.5% of diet items; 7.7% occurrence).

We observed cannibalism in 16 *G*. *affinis* adults (3.2% occurrence), with cannibalized victims accounting for 0.6% of the total prey found in gut contents. Females exhibited greater cannibalism prevalence (3.7%, 15 of 405) than males (1.0%, 1 of 97). In all cases, the cannibalized victim was a small juvenile that had apparently been delivered during the experiment. Cannibals spanned a large range of body size, from 17.7 to 44.1 mm SL (mean = 27.7 mm SL), which covered much of the total range observed in the 502 adults recovered at the end of the experiment within the 34 mesocosms included in cannibalism analyses (14.7–46.9 mm SL).

**FIGURE 5:**
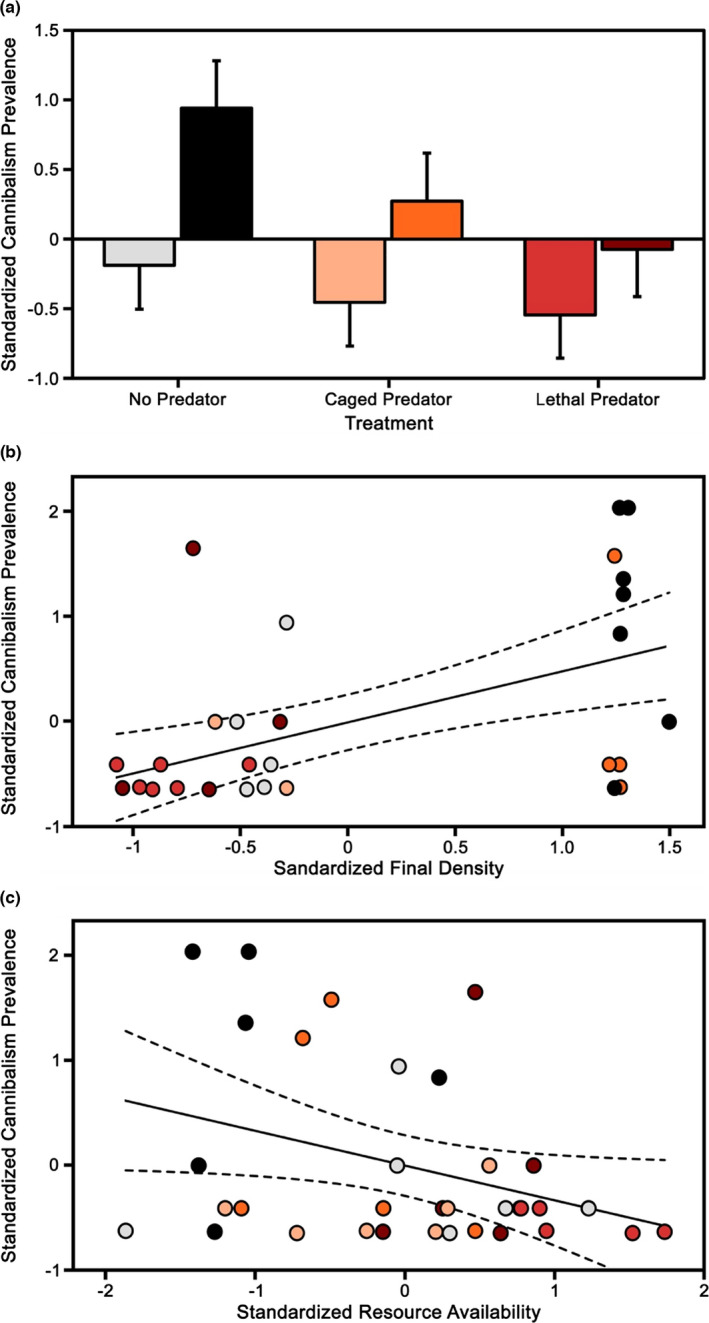
Cannibalism in the *Gambusia affinis* mesocosm experiment. (a) Mean and SE for standardized cannibalism prevalence (i.e., total number of cannibalism occurrences divided by the number of adults with food items in their guts) across the three different predator treatments. Within each predator treatment, left bars represent low‐density treatments and right bars high‐density treatments. Significant relationships between (b) standardized cannibalism prevalence and standardized final density and (c) standardized cannibalism prevalence and standardized resource availability, with best‐fit line and 95% confidence intervals. Colors of symbols in (b) and (c) follow (a)

ANOVA revealed that cannibalism was more frequent within the high‐density treatment (*F*_1,28_ = 8.69, *p* = .0064), showed little evidence for a role of predation threat (*F*_2,28_ = 2.42, *p* = .1076), and found no evidence for an interaction between density and predation (*F*_1,28_ = 0.55, *p* = .58; Figure 5a). While cannibalism was positively associated with final adult density (*r* = 0.51, *p* = .0018; Figure 5b), it was not associated with the estimated density of juveniles at the end of the experiment (*r* = 0.16, *p* = .37). In our more direct tests of the impacts of resource competition on cannibalism, we found that cannibalism was negatively associated with resource availability at the end of the experiment (*r* = −0.35, *p* = .0433; Figure 5c), and showed a negative correlation with average growth rate (*r* = −0.34, *p* = .0483).

## 4 DISCUSSION

We conducted an extensive survey of 17 species of poeciliid fishes (11,946 individuals), many of which are well known to exhibit high rates of cannibalism in captive settings. These fish were sampled across 189 populations in the wild, spanning native ranges in North America, Mexico, and the Caribbean, as well as invasive ranges in Hawaii, the Caribbean and Europe, and including disparate habitat types (e.g., ponds, lakes, rivers). In support of our a priori prediction 1a, we found cannibalism was rare in the wild: absent in 14 of the 17 surveyed species, and rare even in the three species (*G*. *manni*, *G*. *holbrooki* and *G*. *hubbsi*) in which we found it. This is in stark contrast to the high cannibalism rates reported from captive settings, experiments and aquaculture (e.g., Baldwin, 1980; Dionne, 1985; Hubbs, 1996; Jones et al., 1998; Naumowicz et al., 2017), but aligns well with previous studies on wild‐caught fish, which have often reported cannibalism rates of around 1% (Crivelli & Boy, 1987; Gluckman & Hartney, 2000; Hubbs, 1971, 1991; Nesbit & Meffe, 1993; Specziár, 2004). It is also worth noting that several previous gut‐content analyses conducted in guppies and *Gambusia*, did not report any incidence of cannibalism (e.g., Bassar et al., 2010; Crivelli & Boy, 1987; Ganassin et al., 2020; Gkenas et al., 2012; Pirroni et al., 2021; Zandonà et al., 2011, 2015). For cannibalism to comprise an important selective agent, it needs to represent an important cause of mortality in nature, as is certainly the case in some taxa (e.g., Balme & Hunter, 2013; Brown et al., 2021; Elgar & Crespi, 1992; Polis, 1981). While cannibalism is clearly part of the natural behavioral repertoire of mosquitofish and guppies, it constitutes a relatively rare event in natural settings, and thus cannibalism probably does not cause much selection on the traits of these poeciliid fish in most natural populations and under most circumstances.

Congruent with our prediction 2, and in agreement with some previous studies (Hubbs, 1991, 1996), cannibals were predominantly females. This probably resulted in part from the sexual size dimorphism in mosquitofishes (Bisazza, 1993; Riesch et al., 2013, 2018), and poeciliid fishes in general (Snelson, 1989), where females are usually larger, on average, than males. Larger individuals might more readily consume conspecifics due to elevated detection or capture success owing to factors such as altered habitat use, increased locomotor performance, larger gape size, or stronger bite force. Previous work on teleost fishes (Pereira et al., 2017), amphibians (Nyman et al., 1993; Pizzatto & Shine, 2008), spiders (Wilder & Rypstra, 2008), insects (Richardson et al., 2010), and mammals (i.e., infanticide; Lukas & Huchard, 2014) has shown that the prevalence of cannibalism under natural and experimental conditions often increases with increasing size heterogeneity, resulting in large individuals preying on small individuals. In line with this argument, cannibals were substantially larger than their victims also in our study. Size at birth in *Gambusia* species and guppies typically ranges from about 6 to 11 mm SL (e.g., Bashey, 2008; Krumholz, 1948; O’Dea et al., 2015; Wischnath, 1993). Recent work in *G*. *hubbsi* inhabiting blue holes on Andros Island (where most cannibalism observed here occurred) found that size at birth in 8 populations ranged from 9.0 to 10.4 mm SL (Hulthén et al., 2021). While we have observed adult females cannibalizing adult males in captivity (R Riesch & RB Langerhans, personal observation), most cannibalized fish in natural populations appear to be quite young. Given the size range of victims we found in Androsian blue holes (i.e., 9.3 to 15.8 mm SL), these data indicate that some, but not all cannibalized juveniles were newborns. If we assume all victims in Androsian blue holes smaller than 11.0 mm SL were newborns, then half of the measured victims (11 of 22) were newborns.

Yet, if the greater prevalence of cannibalism in females was mostly caused by their larger body size, then we should have predominantly observed cannibalism in larger adults, with body size being an important constraint on cannibalism. On the contrary, we found that the size of cannibalistic individuals varied considerably in mosquitofish, indicating that females (and males) of a wide size range may cannibalize young. The body sizes of cannibalistic individuals we report on here span much of the body size ranges reported for both sexes for these populations in previous studies (*G*. *holbrooki* females: 18.7–37.1 mm SL, *G*. *holbrooki* males: 14.4–25.6 mm SL; *G*. *hubbsi* females: 17.9–47.4 mm SL, *G*. *hubbsi* males: 15.4–35.6 mm SL; *G*. *manni* females: 18.2–42.3 mm SL, *G*. *manni* males: 15.3–30.0 mm SL; Langerhans et al., 2005, 2007, 2009, 2018; Riesch et al., 2013, 2015, 2018). More specifically, the observed body sizes of cannibals spanned approximately 92% of the total range of adult body sizes reported for these populations, and results from our mesocosm experiment were similar, with cannibals spanning 82% of the range of adult body sizes. Assuming that newborns for the focal species generally range from about 6 mm SL to about 11 mm SL (e.g., Bashey, 2008; Krumholz, 1948; O’Dea et al., 2015; Wischnath, 1993), and that a cannibal‐victim size ratio of about 2.0 represents an approximate threshold for cannibalism in these species, then any adults at least twice the size of newborns should be capable of cannibalizing small juveniles. This estimate indicates that most adult *Gambusia* and guppies (except maybe the smallest males) should be capable of cannibalism and implies that the smaller body size of males compared to females unlikely fully explains the lower cannibalism prevalence observed in males. Thus, rather than cannibals mostly comprising large females—at least, larger than most conspecific males—we discovered that adults of virtually any size might exhibit cannibalism, with larger individuals tending to consume larger victims. Even if we exclude all large, cannibalistic females outside the size range of males in this study, we still find that females exhibited cannibalism more than twice as frequently as males overall (0.46% vs. 0.20%). Some additional factor(s), therefore, must be important in explaining why females exhibited a higher prevalence of cannibalism than males in natural populations of mosquitofish.

We propose that female poeciliids of these species tend to have higher energy requirements than males, and thus females have likely experienced stronger selection on energy‐acquisition behaviors. First, females generally have a greater investment into reproductive tissue than males (i.e., ovaries and oocytes/embryos vs testis; Hayward & Gillooly, 2011; Riesch et al., 2011, 2013, 2015, 2016, 2018). Poeciliid females also bear live young, which have high energy demands themselves, but additionally impose costs during pregnancy in the forms of reduced swimming performance and increased oxygen consumption (e.g., Banet et al., 2016; Boehlert et al., 1991; Ghalambor et al., 2004; Plaut, 2002; Quicazan‐Rubio et al., 2019; Srean et al., 2017; Timmermann & Chapman, 2003)—these costs can be mitigated with increased energy intake. These females additionally show indeterminate growth, reaching larger body sizes than males which essentially stop growing after sexual maturity (Bisazza, 1993; Riesch et al., 2013, 2018; Snelson, 1989). Moreover, because larger females typically have larger broods, females may have greater motivation for gathering resources than males since growth can increase fitness (e.g., Auer et al., 2010; Barneche et al., 2018; Hulthén et al., 2021; Riesch et al., 2013, 2018). Consistent with this notion, females often show higher foraging rates than males in the wild (Heinen et al., 2013; Magurran, 2005) and can show higher foraging and food consumption rates in populations with stronger resource competition (Pärssinen et al., 2021). Altogether, it seems that the elevated benefits in females of feeding on high‐energy prey may partially explain the increased cannibalism observed in female mosquitofish.

Both correlative evidence in Bahamas mosquitofish and experimental evidence in Western mosquitofish provided support for our prediction 1b (resource competition), but not for our prediction 3 (predation). In the wild, evidence from tidal creeks was weak: we observed higher cannibalism prevalence in fragmented tidal creeks, as expected based on the higher population densities, greater resource competition, and lower predation risk in these sites (e.g., Araújo et al., 2014; Riesch et al., 2015), but occurrence was so low (1 of 46 populations) that we cannot draw any strong conclusions from this pattern. However, patterns from natural blue‐hole populations were more distinct: we only observed cannibalism in low‐predation blue holes, not in any blue hole with predatory fish (supporting prediction 3). While this suggests a role of predation risk in driving cannibalism behavior, low‐predation populations also tended to have higher population densities, and thus stronger resource competition, than high‐predation populations (e.g., Heinen et al., 2013; Riesch et al., 2020). Patterns observed within low‐predation blue holes—where the prevalence of cannibalism increased with one estimate of the intensity of resource competition (population density) but was unassociated with estimates of encounter rates with juveniles—suggests that resource competition, not predation risk per se, may largely underlie these patterns (supporting prediction 1b). In fact, population density alone could statistically explain the lack of cannibalism observed in high‐predation blue holes in *G*. *hubbsi*: our regression analyses within low‐predation localities predicted that populations with a density below ~2.0 fish per m^3^ should exhibit no cannibalism—all high‐predation populations met this criterion, while no low‐predation population did. Our mesocosm experiment with *G*. *affinis* further strengthened this interpretation, as cannibalism prevalence increased with higher conspecific density, lower resource levels, and lower growth rates, but was unassociated with juvenile density (supporting prediction 1b) and not strongly influenced by predation risk (contrary to prediction 3). In the experiment, the threat of predation *per se* had little effect on cannibalism prevalence, while the indirect effects of predation via reduced density and elevated resource availability did apparently reduce the likelihood of cannibalism (e.g., see the especially low cannibalism prevalence within the lethal predator treatment characterized by particularly low densities and high resource availability, Figure 5b,c). Our findings are therefore congruent with optimal foraging theory, which posits that the optimal diet should be dependent on the energetic returns of a diet item (i.e., benefits) when weighed against the costs involved in finding, capturing, handling and consuming the diet item (MacArthur & Pianka, 1966; Pyke, 1984; Schoener, 1971; Stephens & Krebs, 1986). In other words, cannibalism should become a viable option for resource acquisition when competition for other resources is particularly strong, as it is under high population density. Meanwhile, the putative costs of cannibalism associated with increased vulnerability to predation seem to be of comparatively minor importance. Considering the widespread applicability of optimal foraging theory, and the general importance of resource competition in shaping foraging behaviors in animals (e.g., Ferretti et al., 2019; Mitchell et al., 1990; Willis, 1966), our finding that resource competition appears to be the primary driver of cannibalism in mosquitofishes may prove generally applicable to other taxa. However, it is important to note that we cannot fully disentangle the role of research competition from other possible effects of density in all instances.

Regarding whether or not kin recognition might partially regulate cannibalism, we found support for our prediction 4, as cannibalism rates were not lower in more dispersal‐limited populations compared to less dispersal‐limited populations. Specifically, cannibalism prevalence was not lower in fragmented compared to unfragmented Bahamian tidal creeks nor was it lower in inland blue holes compared to other habitat types in The Bahamas. If mosquitofishes lacked kin recognition, we might have expected more dispersal‐limited populations to show reduced cannibalism because of the higher potential of cannibalizing close relatives (Boots et al., 2021; Lion & van Baalen, 2007; Rudolf et al., 2010). This reinforces the notion that poeciliids can readily discriminate kin from non‐kin (e.g., Greenway et al., 2016; Hain et al., 2017; Langerhans & Makowicz, 2013; Loekle et al., 1982) and can thus potentially avoid cannibalizing close relatives and reduce this possible fitness cost of cannibalism (Pfennig & Collins, 1993; Pfennig et al., 1993). Future experiments providing kin and non‐kin offspring as potential prey (i.e., using protocols similar to Pfennig & Collins, 1993 and Pfennig et al., 1993) could investigate this further.

Lastly, could it be that we mistakenly took interspecific predation for cannibalism in locations where our focal species co‐occurred with another poeciliid species? We do not find this likely for two reasons. First, we never found a case of cannibalism within populations coexisting with a congener. Second, in populations where our focal species co‐occurred with another poeciliid (e.g., *Gambusia holbrooki* co‐occurred in Florida with *Poecilia latipinna* at Panacea Mineral Springs and Ditch off Hwy 98; Table S2), we could identify the victims as conspecifics in all cases (based on external characteristics following dissections).

In conclusion, cannibalism in wild mosquitofish and guppies is rare, probably at least in part because conspecific individuals represent energetically costly prey (i.e., large and highly evasive relative to typical prey) that become worth the effort only when competition for food is intense. This suggests that cannibalism is unlikely to exert strong selection on phenotypes in most wild populations, except in rare cases when population densities are especially high. Predation risk may weakly influence cannibalism in some cases, but its indirect effects via reduced population density appear much more important. While females show a much higher cannibalism prevalence than males, this is only partially explained by their larger average body size—sex differences in energetic demands are likely important. While quite rare in the wild, cannibalism in captive settings can be much more frequent owing to the artificially reduced costs of capturing conspecifics in the confined and limited aquarium space, so that repeated attempts to capture smaller conspecifics are more readily accomplished. Whether cannibalism‐induced selection in captive settings unwittingly alters phenotypes of captive animals when care is not taken to minimize cannibalism requires future investigation. One example where this would be particularly important is in experimental evolution studies in which small populations of fish are kept in mesocosms for a number of generations under certain conditions and are allowed to evolve under these semi‐natural settings. Our study highlights the utility of leveraging large datasets in the study of rare or difficult‐to‐observe phenomena, and the caution that should be exercised when attempting to infer natural behaviors from observations in captive settings.

## CONFLICT OF INTEREST

None declared.

## AUTHOR CONTRIBUTIONS

**Rüdiger Riesch:** Conceptualization (equal); Data curation (equal); Funding acquisition (equal); Investigation (equal); Methodology (equal); Resources (equal); Supervision (equal); Visualization (lead); Writing – original draft (equal); Writing – review & editing (equal). **Marcio Araújo:** Investigation (supporting); Writing – review & editing (supporting). **Stuart Bumgarner:** Investigation (supporting); Writing – review & editing (supporting). **Caitlynn Filla:** Investigation (supporting); Writing – review & editing (supporting). **Laura Pennafort:** Investigation (supporting); Writing – review & editing (supporting). **Taylor Goins:** Investigation (supporting); Writing – review & editing (supporting). **Darlene Lucion:** Investigation (supporting); Writing – review & editing (supporting). **Amber Makowicz:** Investigation (supporting); Writing – review & editing (supporting). **Ryan Martin:** Investigation (supporting); Writing – review & editing (supporting). **Sara Pirroni:** Investigation (supporting); Writing – review & editing (supporting). **Brian Langerhans:** Conceptualization (equal); Data curation (equal); Formal analysis (lead); Funding acquisition (equal); Investigation (equal); Methodology (equal); Resources (equal); Supervision (equal); Visualization (supporting); Writing – original draft (equal); Writing – review & editing (equal).

## Supporting information

Supplementary Material

## Data Availability

All data needed to perform the presented analyses are available in the associated tables and on Figshare (doi: https://doi.org/10.17637/rh.19322927).
