# Supplementary material for: Resource competition explains rare cannibalism in the wild in livebearing fishes
Source: Ecol Evol. 2022 May 16;12(5):e8872. doi: 10.1002/ece3.8872 (PMC9109233; doi:10.1002/ece3.8872)
Supplement: Supplementary file 1 — Supplementary Material [file ECE3-12-e8872-s001.docx]

**Appendix A**

**Table A1.** Sample locations, year of sampling, sample sizes, occurrence of cannibalism, and method of assessment (XR: x-ray imaging, LH: life-history dissections, DI: diet analysis of stomach contents) for adult males and females of 11 species of *Gambusia*, and for *Heterophallus milleri* and *Poecilia reticulata*. If no number precedes the method of assessment, then all specimens were examined using that method (multiple methods could be used per specimen).

| Species | Population | Year | Latitude | Longitude | Cannibalism by Males | Cannibalism by Females | Prevalence of Cannibalism [%] | Method of  Assessment |
| --- | --- | --- | --- | --- | --- | --- | --- | --- |
| *Gambusia affinis* | Travertine Creek, OK (native) | 2009 | 34.50414 | -96.97130 | 0 / 11 | 0 / 61 | 0 | LH |
| N = 410 | Vendome Well, OK (native) | 2009 | 34.50591 | -96.97209 | 0 / 46 | 0 / 56 | 0 | LH |
|  | Zoddletone Creek, OK (native) | 2009 | 35.00455 | -98.68930 | 0 / 21 | 0 / 62 | 0 | LH |
|  | Keahala Mid, Hawaii (invasive) | 2011 | 21.41553 | -157.8107 | 0 / 31 | 0 / 70 | 0 | XR |
|  | Waimanalo Low, Hawaii (invasive) | 2011 | 21.35290 | -157.7241 | 0 / 11 | 0 / 41 | 0 | XR |
|  |  |  |  |  |  |  |  |  |
| *Gambusia eurystoma* | Baños del Azufre, Mexico (native) | 2010 | 17.55256 | -92.99763 | 0 / 27 | - | 0 | LH |
| N = 89 | Baños del Azufre, Mexico (native) | 2009 |  |  | - | 0 / 20 | 0 | LH |
|  | Baños del Azufre, Mexico (native) | 2008 |  |  | 0 / 7 | 0 / 35 | 0 | LH |
|  |  |  |  |  |  |  |  |  |
| *Gambusia geiseri* | Spring Lake, San Marcos, TX (native) | 2006 | 29.89223 | -97.93203 | 0 / 42 | 0 / 90 | 0 | XR |
| N = 169 | San Marcos River, TX (native) | 2006 | 29.87545 | -97.93214 | 0 / 2 | 0 / 35 | 0 | XR |
|  |  |  |  |  |  |  |  |  |
| *Gambusia melapleura* | Bluefields, Jamaica (native) | 2005 | 18.17126 | -78.02353 | 0 / 7 | 0 / 40 | 0 | XR |
| N = 47 |  |  |  |  |  |  |  |  |
|  |  |  |  |  |  |  |  |  |
| *Gambusia panuco* | Arroyo del Encino, Tamaulipas, Mexico (native) | 2005 | 23.13978 | -99.11427 | 0 / 20 | 0 / 38 | 0 | XR |
| N = 58 |  |  |  |  |  |  |  |  |
|  |  |  |  |  |  |  |  |  |
| *Gambusia puncticulata* | Sea Pond, Grand Cayman Island (native) | 2006 | 19.39493 | -81.38380 | 0 / 2 | 0 / 8 | 0 | XR |
| N = 546 | Northeast Pond, Grand Cayman Island (native) | 2006 | 19.35039 | -81.09656 | 0 / 5 | 0 / 52 | 0 | XR, 23 LH, 5 DI |
|  | Bird Pond, Grand Cayman Island (native) | 2006 | 19.34011 | -81.08694 | 0 / 12 | 0 / 34 | 0 | XR, 12 LH, 13 DI |
|  | Tarpon Pond, Grand Cayman Island (native) | 2006 | 19.38729 | -81.37562 | 0 / 16 | 0 / 112 | 0 | XR, 33 LH |
|  | Sinkhole, Little Cayman Island (native) | 2006 | 19.70938 | -79.96897 | 0 / 31 | 0 / 63 | 0 | XR |
|  | Lighthouse Pond, Little Cayman Island (native) | 2006 | 19.65902 | -80.10854 | 0 / 5 | 0 / 28 | 0 | XR, 5 LH, 5 DI |
|  | Tarpon Lake, Little Cayman Island (native) | 2006 | 19.67704 | -80.03941 | 0 / 2 | 0 / 29 | 0 | XR, 2 LH, 4 DI |
|  | Red Shrimp Hole, Cayman Brac (native) | 2006 | 19.69355 | -79.84763 | 0 / 4 | 0 / 31 | 0 | XR |
|  | Coral Isle Pond, Cayman Brac (native) | 2006 | 19.68724 | -79.86536 | 0 / 27 | 0 / 85 | 0 | XR, 80 LH, 42 DI |
|  | Port Royal, Jamaica (native) | 2005 | 17.93611 | -76.80617 | 0 / 23 | 0 / 30 | 0 | XR |
|  | Port Antonio, Jamaica (native) | 2005 | 18.18251 | -76.46074 | 0 / 10 | 0 / 19 | 0 | XR |
|  |  |  |  |  |  |  |  |  |
| *Gambusia quadruncus* | Rio Guayalejo at El Limon, Tamaulipas, Mexico (native) | 2005 | 22.83158 | -99.01072 | 0 / 16 | 0 / 12 | 0 | XR |
| N = 56 | Ciudad Mante, Tamaulipas, Mexico (native) | 2005 | 22.72524 | -98.95582 | 0 / 8 | 0 / 14 | 0 | XR |
|  | Rio Guayalejo near Llera, Tamaulipas, Mexico (native) | 2005 | 23.31434 | -99.00267 | 0 / 2 | 0 / 3 | 0 | XR |
|  | Laguna de Chairel, Tampico, Tamaulipas, Mexico (native) | 2005 | 22.25065 | -97.88803 | - | 0 / 1 | 0 | XR |
|  |  |  |  |  |  |  |  |  |
| *Gambusia rhizophorae* | Matheson Hammock, FL (native) | 2005 | 25.67862 | -80.26219 | 0 / 4 | 0 / 27 | 0 | XR |
| N = 68 | Key West, FL (native) | 2005 | 24.55800 | -81.77118 | 0 / 20 | 0 / 17 | 0 | XR |
|  |  |  |  |  |  |  |  |  |
| *Gambusia sexradiata* | Mogote del Puyacatengo, Mexico (native) | 2009 | 17.58219 | -92.89986 | 0 / 10 | 0 / 40 | 0 | LH |
| N = 125 | Rio Teapao, Mexico (native) | 2009 | 17.55692 | -92.95098 | 0 / 22 | 0 / 53 | 0 | LH |
|  |  |  |  |  |  |  |  |  |
| *Gambusia vittata* | Nacimiento del Rio Mante, Tamaulipas, Mexico (native) | 2005 | 22.69919 | -99.04552 | 0 / 7 | 0 / 38 | 0 | XR |
| N = 45 |  |  |  |  |  |  |  |  |
|  |  |  |  |  |  |  |  |  |
| *Gambusia wrayi* | Middle Quarters, Jamaica (native) | 2005 | 18.10438 | -77.82636 | 0 / 17 | 0 / 28 | 0 | XR |
| N = 58 | Mearnsville, Jamaica (native) | 2005 | 18.19194 | -78.03802 | 0 / 8 | 0 / 5 | 0 | XR |
|  |  |  |  |  |  |  |  |  |
| *Heterophallus milleri* | Arroyo Bonita, Mexico (native) | 2010 | 17.42706 | -92.75194 | 0 / 23 | 0 / 52 | 0 | LH |
| N = 75 |  |  |  |  |  |  |  |  |
|  |  |  |  |  |  |  |  |  |
| *Poecilia reticulata* | Pitch Lake, Trinidad (native) | 2012 | 10.23405 | -61.62714 | 0 / 8 | 0 / 18 | 0 | LH |
| N = 477 | Reference Ditch, Trinidad (native) | 2012 | 10.23770 | -61.61983 | 0 / 21 | 0 / 25 | 0 | LH |
|  | Pitch Lake, Trinidad (native) | 2018 | 10.23405 | -61.62714 | 0 / 11 | 0 / 9 | 0 | LH |
|  | La Brea Village, Trinidad (native) | 2018 | 10.23730 | -61.61402 | 0 / 10 | 0 / 10 | 0 | LH |
|  | Southern Main Road, Trinidad (native) | 2018 | 10.20165 | -61.63299 | 0 / 12 | 0 / 8 | 0 | LH |
|  | Ditch near Vance River, Trinidad (native) | 2018 | 10.19852 | -61.62986 | 0 / 10 | 0 / 10 | 0 | LH |
|  | Downstream Vance River, Trinidad (native) | 2018 | 10.19643 | -61.63309 | 0 / 10 | 0 / 10 | 0 | LH |
|  | Dehli's Grant extension, Trinidad (native) | 2018 | 10.15492 | -61.67229 | 0 / 10 | 0 / 10 | 0 | LH |
|  | Roussilac, Trinidad (native) | 2018 | 10.19931 | -61.59688 | 0 / 10 | 0 / 10 | 0 | LH |
|  | Broken Bridge Forest Reserve, Trinidad (native) | 2018 | 10.16997 | -61.56715 | 0 / 10 | 0 / 10 | 0 | LH |
|  | Parry Lands to Point Fortin, Trinidad (native) | 2018 | 10.17332 | -61.64029 | 0 / 10 | 0 / 10 | 0 | LH |
|  | Point Fortin, Trinidad (native) | 2018 | 10.17080 | -61.68112 | 0 / 10 | 0 / 10 | 0 | LH |
|  | Arima Valley, Trinidad (native) | 2018 | 10.68916 | -61.29010 | 0 / 11 | 0 / 9 | 0 | LH |
|  | Waikone High, Oahu, Hawaii (invasive) | 2011 | 21.49701 | -157.86566 | 0 / 15 | 0 / 15 | 0 | XR |
|  | Keahala High, Oahu, Hawaii (invasive) | 2011 | 21.41434 | -157.81602 | 0 / 21 | 0 / 32 | 0 | XR |
|  | Manoa High, Oahu, Hawaii (invasive) | 2011 | 21.32813 | -157.80103 | 0 / 41 | 0 / 61 | 0 | XR |
| Total |  |  |  |  | 0 / 719 | 0 / 1586 | 0 |  |

**Table A2.** Sample locations, year of sampling, sample sizes, occurrence of cannibalism, and method of assessment (XR: x-ray imaging, LH: life-history dissections, DI: diet analysis of stomach contents) for adult males and females of *Gambusia holbrooki*.

| Population | Year | Latitude (N) | Longitude (W) | Cannibalism by Males | Cannibalism by Females | Prevalence of Cannibalism [%] | Method of Assessment |
| --- | --- | --- | --- | --- | --- | --- | --- |
| *a) Native range:* |  |  |  |  |  |  |  |
| Lake Lilly, NJ | 2011 | 38.93829 | -74.96416 | 0 / 5 | 0 / 35 | 0 | LH |
| Rehoboth Beach, DE | 2011 | 38.71799 | -75.08268 | 0 / 11 | 0 / 30 | 0 | LH |
| Suffolk, VA | 2011 | 36.89134 | -76.44301 | 0 / 9 | 0 / 30 | 0 | LH |
| Ditch along Hwy 45, NC | 2011 | 35.58629 | -76.50341 | 0 / 20 | 2 / 31 | 3.9 | LH |
| Charleston, SC | 2011 | 32.73412 | -79.99592 | 0 / 20 | 0 / 30 | 0 | LH |
| Ditch outside St Simon, GA | 2011 | 32.73412 | -79.99592 | 0 / 20 | 1 / 20 | 2.5 | LH |
| Daytona Beach, FL | 2011 | 29.22824 | -81.02786 | 0 / 20 | 0 / 26 | 0 | LH |
| City Pond, FL | 2011 | 28.14445 | -80.59733 | 0 / 45 | 0 / 103 | 0 | LH |
| Port Lucie, FL | 2011 | 27.29347 | -80.30026 | 0 / 16 | 0 / 24 | 0 | LH |
| Big Pine Key, FL | 2010 | 24.69510 | -81.37943 | 0 / 28 | 0 / 49 | 0 | XR |
| Fort Zachary Taylor SP, FL | 2011 | 24.54694 | -81.80953 | 0 / 21 | 0 / 6 | 0 | LH |
| Green Springs, FL | 2011 | 28.86307 | -81.24856 | 0 / 29 | 0 / 29 | 0 | LH |
|  | 2012 |  |  | 0 / 23 | 0 / 58 | 0 | LH |
| Lake Monroe, FL | 2011 | 28.86233 | -81.25275 | 0 / 26 | 0 / 68 | 0 | LH |
|  | 2012 |  |  | 0 / 20 | 0 / 37 | 0 | LH |
| Newport Springs, FL | 2011 | 30.21271 | -84.17857 | 0 / 36 | 0 / 30 | 0 | LH |
|  | 2012 |  |  | 0 / 24 | 0 / 49 | 0 | LH |
| Ditch in St Marks, FL | 2012 | 30.15457 | -84.20542 | 0 / 24 | 0 / 44 | 0 | LH |
| Panacea Mineral Springs, FL | 2011 | 30.03448 | -84.38982 | 0 / 13 | 0 / 35 | 0 | LH |
|  | 2012 |  |  | 0 / 12 | 1 / 66 | 1.3 | LH |
| Ditch off Hwy 98, FL | 2011 | 29.79787 | -84.74463 | 0 / 10 | 0 / 0 | 0 | LH |
|  | 2012 |  |  | 0 / 18 | 1 / 35 | 1.9 | LH |
| *b) Invasive range:* |  |  |  |  |  |  |  |
| Nassau, The Bahamas | 2010 | 24.99735 | -77.35915 | 0 / 34 | 0 / 78 | 0 | XR |
| Lago di Fimon North, Italy | 2017 | 45.47080 | 11.54077 | 0 / 6 | 0 / 10 | 0 | DI |
| Lago di Fimon South, Italy | 2017 | 45.46343 | 11.54237 | 0 / 9 | 0 / 10 | 0 | DI |
| Marina di Grosseto, Italy | 2017 | 42.73109 | 10.96338 | 0 / 13 | 0 / 6 | 0 | DI |
| E of Marina di Grosseto, Italy | 2017 | 42.73347 | 11.04127 | 0 / 13 | 0 / 10 | 0 | DI |
| El Palmar, Spain | 2017 | 39.31164 | -0.32049 | 0 / 8 | 0 / 10 | 0 | DI |
| Rio Xuquer, Spain | 2017 | 39.17749 | -0.26924 | 0 / 10 | 0 / 10 | 0 | DI |
| Rio Vaca, Spain | 2017 | 39.06061 | -0.21827 | 0 / 10 | 0 / 10 | 0 | DI |
| Lebrija, Spain | 2017 | 36.96006 | -6.06446 | 0 / 6 | 0 / 10 | 0 | DI |
| N of Doñana, Spain | 2017 | 37.20174 | -6.26175 | 0 / 2 | 0 / 10 | 0 | DI |
| Total |  |  |  | 0 / 499 | 5 / 872 | 0.4 |  |

**Table A3.** Sample locations, year of sampling, sample sizes, occurrence of cannibalism, and method of assessment (XR: x-ray imaging, LH: life-history dissections, DI: diet analysis of stomach contents) for native populations of three different species of *Gambusia* from The Bahamas. TC: tidal creek, BH: blue hole, P: pond. If no number precedes the method of assessment, then all specimens were examined using that method (multiple methods could be used per specimen).

| Species | Island | Habitat | Population | Year | Latitude | Longitude | Cannibalism  by Males | Cannibalism  by Females | Prevalence of Cannibalism [%] | Method of Assessment |
| --- | --- | --- | --- | --- | --- | --- | --- | --- | --- | --- |
| *Gambusia sp.* | Abaco | TC | Loggerhead Creek | 2009 | 26.5967 | -77.1819 | 0 / 3 | 0 / 8 | 0 | DI |
| N = 1,998 |  | TC |  | 2010 |  |  | 0 / 20 | 0 / 34 | 0 | 41 XR, 13 DI |
|  |  | TC | Crossing Rocks | 2005 | 26.1488 | -77.1899 | 0 / 2 | 0 / 0 | 0 | XR |
|  |  | TC |  | 2006 |  |  | 0 / 3 | 0 / 10 | 0 | XR |
|  |  | TC |  | 2009 |  |  | 0 / 6 | 0 / 13 | 0 | DI |
|  |  | TC |  | 2010 |  |  | 0 / 5 | 0 / 12 | 0 | 5 XR, 12 DI |
|  |  | TC | Double Blocked Down | 2009 | 26.6090 | -77.2577 | 0 /6 | 0 / 13 | 0 | DI |
|  |  | TC |  | 2010 |  |  | 0 / 26 | 0 / 47 | 0 | 60 XR, 17 LH, 13 DI |
|  |  | TC | Double Blocked Up | 2009 | 26.6091 | -77.2577 | 0 / 9 | 0 / 24 | 0 | DI |
|  |  | TC |  | 2010 |  |  | 0 / 27 | 0 / 40 | 0 | 53 XR, 10 LH, 14 DI |
|  |  | TC | Indian River East | 2005 | 26.2919 | -77.1124 | 0 / 23 | 0 / 35 | 0 | XR |
|  |  | TC |  | 2009 |  |  | 0 / 6 | 0 / 19 | 0 | 5 XR, 20 DI |
|  |  | TC |  | 2010 |  |  | 0 / 29 | 0 / 41 | 0 | 45 XR, 20 LH, 25 DI |
|  |  | TC | Sandy Point | 2005 | 26.0075 | -77.4035 | 0 / 63 | 0 / 44 | 0 | XR |
|  |  | TC |  | 2009 |  |  | 0 / 38 | 0 / 52 | 0 | DI |
|  |  | TC |  | 2010 |  |  | 0 / 19 | 0 / 32 | 0 | 39 XR, 20 LH, 12 DI |
|  |  | TC | Stinky Pond | 2005 | 26.5821 | -77.1693 | 0 / 39 | 0 / 74 | 0 | XR |
|  |  | TC |  | 2009 |  |  | 0 / 6 | 0 / 5 | 0 | DI |
|  |  | TC |  | 2010 |  |  | 0 / 40 | 0 / 62 | 0 | 89 XR, 10 LH, 13 DI |
|  |  | TC | Blue Holes Creek | 2009 | 26.3172 | -77.0381 | 0 / 7 | 0 / 6 | 0 | DI |
|  |  | TC |  | 2010 |  |  | 0 / 18 | 0 / 16 | 0 | 22 XR, 22 LH, 12 DI |
|  |  | TC | Cherokee Creek | 2005 | 26.2827 | -77.0425 | 0 / 17 | 0 / 19 | 0 | XR |
|  |  | TC |  | 2009 |  |  | 0 / 3 | 0 / 15 | 0 | DI |
|  |  | TC |  | 2010 |  |  | 0 / 15 | 0 / 20 | 0 | 23 XR, 10 LH, 12 DI |
|  |  | TC | Cross Harbour | 2005 | 25.9563 | -77.2751 | 0 / 27 | 0 / 47 | 0 | XR |
|  |  | TC | Indian River West | 2010 |  |  | 0 / 18 | 0 / 36 | 0 | 40 XR, 14 DI |
|  |  | TC | Sand Bar | 2009 | 26.2800 | -77.0531 | 0 / 30 | 0 / 26 | 0 | DI |
|  |  | TC |  | 2010 |  |  | 0 / 7 | 0 / 24 | 0 | 18 XR, 15 LH, 13 DI |
|  |  | TC | Treasure Cay | 2009 | 26.6838 | -77.3068 | 0 / 5 | 0 / 16 | 0 | DI |
|  |  | TC |  | 2010 |  |  | 0 / 25 | 0 / 32 | 0 | 45 XR, 10 LH, 12 DI |
|  |  | TC | Twisted Bridge | 2009 | 26.6009 | -77.1757 | 0 / 3 | 0 / 12 | 0 | DI |
|  |  | TC |  | 2010 |  |  | 0 / 36 | 0 / 56 | 0 | 80 XR, 20 LH, 12 DI |
|  |  | P | Leisure Lee | 2005 | 26.6178 | -77.2573 | 0 / 15 | 0 / 28 | 0 | XR |
|  |  | BH | Robert's | 2005 | 26.8537 | -77.4896 | 0 / 95 | 0 / 96 | 0 | XR |
|  | Grand Bahama | TC | Crumbling Road | 2010 | 26.6774 | -78.0676 | 0 / 25 | 0 / 46 | 0 | XR, 10 LH |
|  |  | TC | Jellyshell West | 2010 | 26.5616 | -78.8433 | 0 / 13 | 0 / 37 | 0 | XR, 16 LH |
|  |  | TC | Rainy Blocked | 2010 | 26.6552 | -78.2817 | 0 / 18 | 0 / 22 | 0 | XR, 10 LH |
|  |  | TC | Blue Holes Creek | 2010 | 26.6590 | -77.9982 | 0 / 16 | 0 / 18 | 0 | XR, 10 LH |
|  |  | TC | Empty House | 2010 | 26.6454 | -77.9340 | 0 / 15 | 0 / 18 | 0 | XR, 10 LH |
|  |  | TC | Expansive Creek | 2010 | 26.6022 | -78.8538 | 0 / 27 | 0 / 38 | 0 | XR, 20 LH |
| *Gambusia hubbsi* | Andros | TC | Fresh Creek Back Up | 2010 | 24.7135 | -78.0662 | 0 / 13 | 0 / 35 | 0 | XR, 20 LH, 14 DI |
| N = 2,264 |  | TC | Independence Park | 2002 | 24.4674 | -77.7309 | - | 0 / 16 | 0 | XR, 10 DI |
|  |  | TC |  | 2004 |  |  | 0 / 2 | 0 / 14 | 0 | XR |
|  |  | TC |  | 2010 |  |  | 2 / 14 | 0 / 7 | 9.5 | XR, 10 LH |
|  |  | TC | Red Bays | 2010 | 25.1332 | -78.2056 | 0 / 15 | 0 / 19 | 0 | XR, 16 LH, 3 DI |
|  |  | TC | Thompson/Scott | 2002 | 24.9088 | -77.9355 | 0 / 19 | 0 / 12 | 0 | XR, 30 LH |
|  |  | TC |  | 2004 |  |  | 0 / 22 | 0 / 36 | 0 | XR |
|  |  | TC |  | 2010 |  |  | 0 / 28 | 0 / 88 | 0 | XR, 10 LH, 4 DI |
|  |  | TC |  | 2012 |  |  | 0 / 9 | 0 / 33 | 0 | XR |
|  |  | TC | Cargill Creek | 2010 | 24.4818 | -77.7236 | 0 / 20 | 0 / 33 | 0 | XR, 20 LH |
|  |  | TC | Conch Sound | 2005 | 25.1153 | -78.0034 | 0 / 21 | 0 / 16 | 0 | XR |
|  |  | TC | Davey Creek | 2010 | 25.1479 | -78.0654 | 0 / 14 | 0 / 10 | 0 | XR, 10 LH, 10 DI |
|  |  | TC | Davis Creek | 2004 | 24.7471 | -77.8114 | 0 / 26 | 0 / 40 | 0 | XR |
|  |  | TC | Fresh Creek Twin Lakes | 2010 | 24.7187 | -78.0015 | 0 / 20 | 0 / 30 | 0 | XR, 20 LH, 4 DI |
|  |  | TC | Mastic Point | 2004 | 25.0439 | -77.9789 | 0 / 11 | 0 / 14 | 0 | XR |
|  |  | TC | Stafford Creek North | 2010 | 24.9044 | -77.93496 | 0 / 22 | 0 / 20 | 0 | XR, 10 LH, 2 DI |
|  |  | P | Blood Pond | 2004 | 24.7763 | -77.8560 | 0 / 17 | 0 / 48 | 0 | XR |
|  |  | P | Dredged Pond near Fresh Creek | 2004 | 24.7116 | -77.7971 | 0 / 43 | 0 / 73 | 0 | XR |
|  |  | P | Fowler's Pond | 2002 | 24.9762 | -78.0230 | 0 / 12 | 0 / 36 | 0 | XR |
|  |  | P |  | 2004 |  |  | 0 / 32 | 0 / 84 | 0 | XR |
|  |  | P | London Pond | 2002 | 24.9100 | -77.9844 | 0 / 15 | 1 / 27 | 2.4 | XR, LH |
|  |  | P |  | 2012 |  |  | 0 / 8 | 0 / 32 | 0 | XR, DI |
|  |  | BH | Arieto | 2011 | 25.0634 | -77.9847 | 0 / 30 | 0 / 30 | 0 | XR |
|  |  | BH | Big Blue | 2004 | 24.7897 | -77.8954 | 0 / 13 | 0 / 46 | 0 | XR |
|  |  | BH | Guardian | 2004 | 24.5112 | -77.7212 | 0 / 29 | 0 / 58 | 0 | XR |
|  |  | BH | Long Lonely Hole | 2004 | 24.7587 | -77.9057 | 0 / 3 | 0 / 13 | 0 | XR |
|  |  | BH | Moses Hole | 2004 | 24.7693 | -77.8384 | 0 / 2 | 0 / 26 | 0 | XR |
|  |  | BH | Orchid | 2004 | 24.7899 | -77.9226 | 0 / 10 | 0 / 12 | 0 | XR |
|  |  | BH | Red Bays | 2010 | 25.1466 | -78.1513 | 0 / 27 | 0 / 35 | 0 | XR |
|  | (South Andros) | P | Dump Pond | 2004 | 23.8724 | -77.5168 | 0 / 17 | 0 / 26 | 0 | XR |
|  |  | BH | Batelco | 2010 | 24.0928 | -77.5514 | 0 / 23 | 0 / 20 | 0 | XR |
|  |  | BH | Co-op Hole | 2010 | 24.1081 | -77.5553 | 0 / 21 | 0 / 14 | 0 | XR |
|  |  | BH | Iguana Cenote | 2010 | 24.0826 | -77.7109 | 0 / 31 | 0 / 36 | 0 | XR |
|  |  | BH | Mangrove Lake | 2004 | 24.0742 | -77.5455 | 0 / 22 | 0 / 45 | 0 | XR |
|  |  | BH |  | 2010 |  |  | 0 / 21 | 0 / 24 | 0 | XR |
|  |  | BH | Money Rock | 2004 | 24.0947 | -77.5520 | 0 / 9 | 0 / 34 | 0 | XR |
|  |  | BH |  | 2010 |  |  | 0 / 38 | 0 / 49 | 0 | XR |
|  |  | BH | Nine Tasks | 2010 | 24.0998 | -77.5530 | 0 / 38 | 0 / 38 | 0 | XR |
|  |  | BH | Stargate | 2010 | 24.1062 | -77.5548 | 0 / 13 | 0 / 13 | 0 | XR |
|  |  | BH | Swimming Hole | 2010 | 24.1677 | -77.5872 | 0 / 16 | 0 / 12 | 0 | XR |
|  | New Providence | TC | Adelaide Up | 2004 | 25.0021 | -77.4958 | 0 / 2 | 0 / 18 | 0 | XR |
|  |  | TC |  | 2010 |  |  | 0 / 16 | 0 / 28 | 0 | XR, 20 LH |
|  |  | TC | Foxhill Up | 2010 | 25.0169 | -77.3041 | 0 / 24 | 0 / 31 | 0 | XR, 20 LH |
|  |  | TC | South Beach Up | 2010 | 24.9974 | -77.3592 | 0 / 10 | 0 / 10 | 0 | XR |
|  |  | TC | Defense Creek | 2005 | 24.9903 | -77.4766 | 0 / 2 | 0 / 2 | 0 | XR |
|  |  | TC |  | 2010 |  |  | 0 / 15 | 0 / 31 | 0 | XR, 19 LH |
|  |  | TC | Foxhill Down | 2005 | 25.0116 | -77.3178 | 0 / 3 | 0 / 5 | 0 | XR |
|  |  | TC |  | 2010 |  |  | 0 / 11 | 0 / 13 | 0 | XR, 10 LH |
|  |  | TC | South Beach Creek | 2010 | 25.0031 | -77.3487 | 0 / 22 | 0 / 21 | 0 | XR |
| *Gambusia manni* | Eleuthera | TC | John Miller | 2010 | 24.6922 | -76.1942 | 0 / 14 | 0 / 39 | 0 | XR, 20 LH |
| N = 1,366 |  | TC | Princess Cay | 2010 | 24.6321 | -76.1661 | 0 / 16 | 0 / 31 | 0 | XR, 10 LH |
|  |  | TC | Tarpum Bay | 2010 | 24.9776 | -76.1756 | 0 / 17 | 0 / 46 | 0 | XR, 20 LH |
|  |  | TC | Airport Eleuthera | 2010 | 24.8906 | -76.1660 | 0 / 23 | 0 / 20 | 0 | XR, 20 LH |
|  |  | TC | Cape Eleuthera | 2010 | 24.7844 | -76.3243 | 0 / 15 | 0 / 31 | 0 | XR, 19 LH |
|  |  | TC | Cruise Ship | 2010 | 24.6367 | -76.1687 | 0 / 20 | 0 / 20 | 0 | XR, 20 LH |
|  | Great Exuma | TC | Bahamas Sound Creek | 2005 | 23.5826 | -75.9482 | 0 / 9 | 0 / 11 | 0 | XR |
|  |  | TC | Farmer’s Hill Creek | 2005 | 23.6145 | -75.9121 | 0 / 33 | 0 / 19 | 0 | XR |
|  |  | TC | Saphire Creek | 2005 | 23.6033 | -76.0080 | 0 / 4 | 0 / 8 | 0 | XR |
|  |  | P | Crescent Bay Pond | 2005 | 23.5243 | -75.7927 | 0 / 56 | 0 / 59 | 0 | XR |
|  | (Exuma Cays) | P | Norman's Pond | 2005 | 23.7691 | -76.1309 | 0 / 11 | 0 / 13 | 0 | XR |
|  | Long Island | TC | Airport Creek | 2010 | 23.1642 | -75.1055 | 0 / 17 | 0 / 19 | 0 | XR, 10 LH |
|  |  | TC | Gordon's Beach | 2010 | 22.8706 | -74.8643 | 0 / 50 | 0 / 50 | 0 | XR, 19 LH |
|  |  | TC | Stella Maris | 2010 | 23.5615 | -75.2710 | 0 / 21 | 0 / 43 | 0 | XR, 10 LH |
|  |  | TC | Two Sisters | 2010 | 23.6219 | -75.2913 | 0 / 11 | 0 / 53 | 0 | XR, 16 LH |
|  |  | TC | Clarence Creek | 2010 | 23.0701 | -74.9820 | 0 / 18 | 0 / 20 | 0 | XR, 20 LH |
|  |  | TC | CLIFF Creek | 2010 | 23.1785 | -75.1258 | 0 / 17 | 0 / 20 | 0 | XR, 10 LH |
|  |  | TC | Glintons Creek | 2010 | 23.6524 | -75.3091 | 0 / 12 | 0 / 35 | 0 | XR, 10 LH |
|  |  | TC | Gordon's Creek | 2010 | 22.8835 | -74.8736 | 0 / 15 | 0 / 42 | 0 | XR, 20 LH |
|  |  | BH | Cormorant | 2010 | 23.0046 | -74.8833 | 0 / 32 | 0 / 56 | 0 | XR |
|  |  | BH | Gonzo's | 2010 | 23.6018 | -75.2743 | 0 / 27 | 0 / 33 | 0 | XR |
|  |  | BH | Half House | 2010 | 23.0019 | -74.8869 | 1 / 31 | 1 / 44 | 2.7 | XR |
|  |  | BH | BH 7 | 2010 | 23.0007 | -74.8786 | 0 / 30 | 0 / 29 | 0 | XR |
|  |  | BH | BH 3 | 2010 | 23.0240 | -74.8887 | 0 / 15 | 0 / 36 | 0 | XR |
|  | San Salvador | P | Clear Pond | 2002 | 23.9369 | -74.5493 | 0 / 12 | 1 / 38 | 2.0 | XR |
|  |  | P | Gold Dust Pond | 2002 | 23.9648 | -74.5245 | 0 / 6 | 0 / 19 | 0 | XR |
|  |  |  | Total |  |  |  | 3 / 2188 | 3 / 3440 | 0.1 |  |

**Table A4.** Mean and standard error (in parentheses) of abiotic water conditions measured in the *Gambusia affinis* mesocosm experiment.

| Temperature (°C) | pH | Salinity (ppm) | Conductivity (µS) | DO (mg/L) |
| --- | --- | --- | --- | --- |
| 27.1 (0.18) | 8.6 (0.05) | 282.3 (2.06) | 408.6 (1.79) | 7.92 (0.07) |

**Table A5.** Mean and standard error (in parentheses) of *Gambusia affinis* survival in the mesocosm experiment. LD: low density; HD: high density; NP: no predator; CP: caged predator; LP: lethal predator.

| Density | Predation | Survival |
| --- | --- | --- |
| LD | NP | 100.00% (0.00%) |
| LD | CP | 98.30% (1.70%) |
| LD | LP | 31.70% (7.00% |
| HD | NP | 98.30% (1.10%) |
| HD | CP | 97.30% (1.20%) |
| HD | LP | 22.80% (5.50%) |
